# Functionally characterizing obesity-susceptibility genes using CRISPR/Cas9, in vivo imaging and deep learning

**DOI:** 10.1038/s41598-025-89823-2

**Published:** 2025-02-13

**Authors:** Eugenia Mazzaferro, Endrina Mujica, Hanqing Zhang, Anastasia Emmanouilidou, Anne Jenseit, Bade Evcimen, Christoph Metzendorf, Olga Dethlefsen, Ruth JF Loos, Sara Gry Vienberg, Anders Larsson, Amin Allalou, Marcel den Hoed

**Affiliations:** 1 The Beijer Laboratory, Department of Immunology, Genetics and Pathology, Uppsala University and SciLifeLab, Uppsala , Sweden; 2 Science for Life Laboratory, National Bioinformatics Infrastructure, Stockholm University, Stockholm, Sweden; 3 The Charles Bronfman Institute for Personalized Medicine, Icahn School of Medicine at Mount Sinai, New York, NY 10029 USA; 4 The Mindich Child Health and Development Institute, Icahn School of Medicine at Mount Sinai, New York, NY 10029 USA; 5 Novo Nordisk Foundation Center for Basic Metabolic Research, Faculty of Health and Medical Sciences, University of Copenhagen, Copenhagen, Denmark; 6 Department of Brain and Adipose biology, Måløv, Denmark; 7 Department of Medical Sciences, Clinical Chemistry, Uppsala University, Uppsala , Sweden; 8 Department of Information Technology, Division of Visual Information and Interaction, Uppsala University, Uppsala , Sweden; 9 BioImage Informatics Facility at SciLifeLab, Uppsala, Sweden

**Keywords:** Zebrafish, CRISPR/Cas9, Fluorescence microscopy, Deep learning, Image analysis, Medical genomics, Molecular medicine, Endocrine system and metabolic diseases, Obesity

## Abstract

Hundreds of loci have been robustly associated with obesity-related traits, but functional characterization of candidate genes remains a bottleneck. Aiming to systematically characterize candidate genes for a role in accumulation of lipids in adipocytes and other cardiometabolic traits, we developed a pipeline using CRISPR/Cas9, non-invasive, semi-automated fluorescence imaging and deep learning-based image analysis in live zebrafish larvae. Results from a dietary intervention show that 5 days of overfeeding is sufficient to increase the odds of lipid accumulation in adipocytes by 10 days post-fertilization (dpf, *n* = 275). However, subsequent experiments show that across 12 to 16 established obesity genes, 10 dpf is too early to detect an effect of CRISPR/Cas9-induced mutations on lipid accumulation in adipocytes (*n* = 1014), and effects on food intake at 8 dpf (*n* = 1127) are inconsistent with earlier results from mammals. Despite this, we observe effects of CRISPR/Cas9-induced mutations on ectopic accumulation of lipids in the vasculature (*sh2b1* and *sim1b*) and liver (*bdnf*); as well as on body size (*pcsk1*, *pomca*, *irs1*); whole-body LDLc and/or total cholesterol content (*irs2b* and *sh2b1*); and pancreatic beta cell traits and/or glucose content (*pcsk1*, *pomca*, and *sim1a*). Taken together, our results illustrate that CRISPR/Cas9- and image-based experiments in zebrafish larvae can highlight direct effects of obesity genes on cardiometabolic traits, unconfounded by their – not yet apparent – effect on excess adiposity.

**Supplementary Information:**

The online version contains supplementary material available at 10.1038/s41598-025-89823-2.

## Introduction

Obesity has become a leading cause of health concerns, affecting over 650 million individuals worldwide^1^. Obesity originates from a thermodynamic imbalance between high energy intake and low energy expenditure. Over time, this imbalance leads to the excess storage of triglycerides in lipid droplets^2^. When the limit of the adipose tissue to further expand is exceeded, free fatty acids spill over to ectopic tissues (e.g., pancreas, liver, muscle)^2^, compromising their function. This can lead to comorbidities like type-2 diabetes, steatotic liver disease, and cardiovascular disease^2^. Obesity – typically defined as a body mass index (BMI) > 30 kg/m^2^ – is a complex, multifactorial disease that results from the contribution of environmental and genetic factors.

Our understanding of monogenic forms of obesity largely stems from experiments in murine model systems and from case studies in humans with loss-of-function mutations that cause deficiencies of ligands or their receptors in the leptin-melanocortin pathway. Such genes include leptin receptor (*LEPR*)^3^, melanocortin 4 receptor (*MC4R*)^4,5^, pro-opiomelanocortin (*POMC*)^6^, and proprotein convertase subtilisin/kexin type 1 (*PCSK1*)^7^; as well as downstream actors like SIM bHLH Transcription Factor 1 (*SIM1*) and insulin receptor substrates 1 (*IRS1*)^8^ and 2 (*IRS2*)^9^. In the context of complex obesity, increasingly large genome-wide association studies (GWAS) have identified hundreds of loci that are robustly associated with BMI^10^. While some loci harbor genes with an established role in obesity and body fat distribution, like *MC4R*^11^, *PCSK1*^12^, Brain-derived neurotrophic factor (*BDNF*)^11^, *POMC*, SH2B adaptor protein 1 (*SH2B1*)^11^, and *LEPR*^11^, the role of the vast majority of loci remains unclear and candidate genes have not yet been functionally characterized. Systematic efforts to do so will almost certainly improve our understanding of obesity etiology at a molecular level, and will likely yield targets that can be translated into efficient new medication for prevention and treatment. New, in vivo model systems are desperately needed to accomplish this.

Zebrafish are vertebrate animals with a well-annotated genome that has at least one orthologue for 71.4% of human genes^13^. They possess the key organs^14^, and pathways for body mass control and energy homeostasis are evolutionarily conserved with mammals, including adipocyte differentiation^15^; cholesterol^16^and glucose^17^metabolism; and insulin-like signaling and secretion^18^. Moreover, zebrafish are susceptible to diabetes- and atherosclerosis-like states when exposed to environmental (i.e., dietary interventions) and/or genetic perturbations^19,20^. The zebrafish pancreas shares its morphogenesis and cellular architecture with the mammalian pancreas, with highly conserved signaling pathways and mechanisms^20^. The zebrafish also possesses neuronal circuits controlling lipid storage and appetite and has orthologues of the mammalian ghrelin (*ghrl*), fatty acid binding protein 4a (*fabp4a*), and peroxisome proliferator-activated receptor gamma (*pparg*) genes^21^. They accumulate excess lipids in visceral, intramuscular, and subcutaneous white adipose tissue^15,22^; the energy storage function of adipose tissue has been conserved between fish and mammals^22^; and zebrafish adipocytes express genes associated with adipocyte differentiation^22^, lipolysis^23^, and endocrine function^22,24^. Moreover, zebrafish adipocytes accumulate neutral lipids in multiple small cytoplasmic lipid droplets^22^that – similarly to mammalian white adipose tissue – later converge into single large lipid droplets. Such droplets can be visualized in vivo using lipophilic dyes^25,26^, thanks to the optical transparency of zebrafish larvae during the early stages of development.

Small-scale studies – mainly in adult zebrafish – have shown promising effects on obesity traits for overfeeding^27^, high-fat diet^28^, and genetic manipulation^29^. Overfeeding results in increased adiposity, insulin resistance, hepatic steatosis, and a preferential increase of visceral fat^27,28^. However, adult zebrafish are not an obvious choice for systematic genetic screens. Here, we present and validate a pipeline based on CRISPR/Cas9 ^30,31^; semi-automated positioning, orienting, and fluorescence imaging; deep learning-based image analysis; and individual level biochemistry to explore if zebrafish larvae can be used to systematically functionally characterize genes for a role in obesity and related cardiometabolic traits.

## Results

**Fig. 1:**
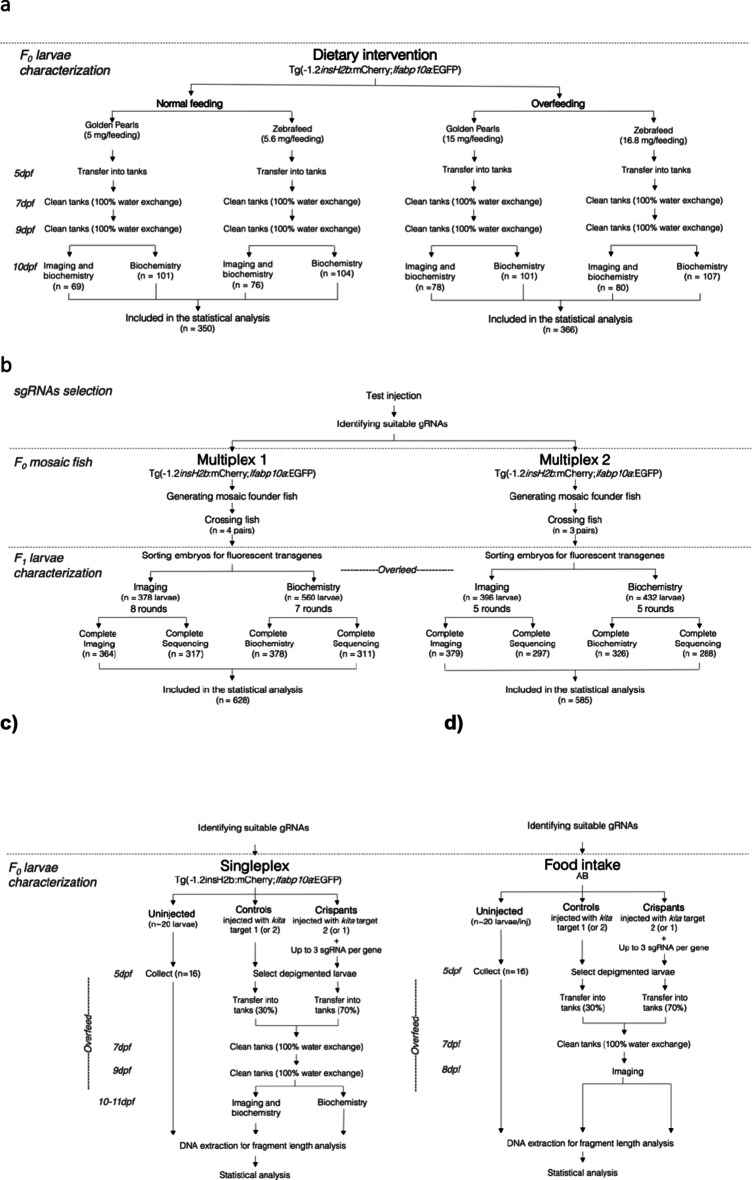
Experimental flow chart. The flow chart describes the workflow, exclusion criteria, and the final number of larvae included in the statistical analysis in all experiments. **(a)** Transgenic (Tg[−1.2*insH2b*:mCherry; 2.8*fabp10a*:GFP]) larvae (AB) were fed on either a control amount (*n* = 350), 3x as much (*n* = 366) from day 5 to day 9 post fertilization. **(b)** Zebrafish orthologues of 12 established human obesity genes were targeted using a multiplexed CRISPR/Cas9 approach. Two sets of CRISPR/Cas9 founders for eight (*arid5b*, *mc4r*, *lepr*, *negr1*, *pcsk1*, *pomca*, *pomcb*, *sec16b*) and seven (*bdnf*,* irs1*,* irs2a*,* irs2b*,* sh2b1*,* sim1a*,* sim1*) orthologues were generated using fertilized eggs from parents with fluorescently labelled liver and pancreatic beta cell nuclei. Founders were raised and in-crossed, and F_1_ larvae were overfed and phenotypically characterized at 10 days post-fertilization (dpf) for 14 traits, using semi-automated fluorescence microscopy and biochemistry. **(c)** Fertilized eggs from parents with fluorescently labelled liver and pancreatic beta cell nuclei were microinjected at the single cell stage with a duplex gRNA targeting *kita* (controls) or additionally with a duplex gRNA to generate affected larvae for the candidate gene of interest. Larvae were overfed from day 5 to day 9. Larvae were imaged on day 10 or 11 and/or collected for biochemistry. **(d)** A duplex gRNA targeting the *kita* gene was injected in fertilized eggs from AB parents to generate controls; while affected larvae were generated by micro-injecting up to three duplex gRNAs targeting early exons or domains of each orthologue of the human gene of interest, alongside the duplex gRNA targeting *kita*. Larvae were overfed from day 5 to day 7. At day 8, larvae were fed with fluorescently labeled food and imaged to quantify food intake.

**Fig. 2:**
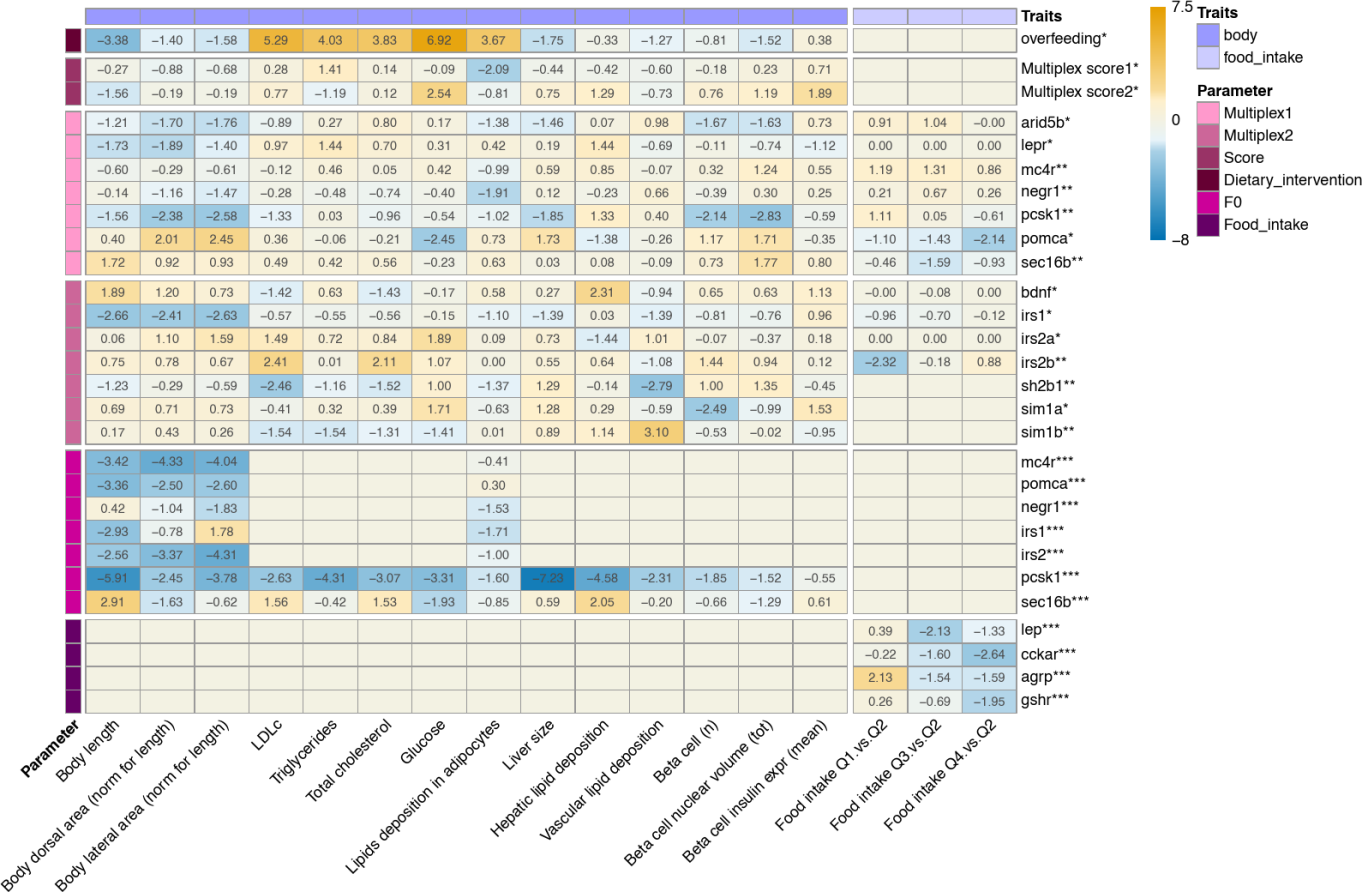
Heatmap visualizing the effects of all genetic and dietary perturbations on obesity-related traits in zebrafish larvae. Numbers in cells are effects of exposure vs. controls expressed in z-scores, so values <−1.96 and > 1.96 reflect effects with *p* < 0.05. The study is divided into five main parts, examining the effect of: (1) overfeeding in larvae free from CRISPR/Cas9-induced mutations; (2) genetic burden scores comprising the summed number of mutated alleles across the eight (Multiplex score 1) and seven (Multiplex score 2) genes targeted simultaneously. Mutations in each allele are weighted by their predicted effect on protein function. Larvae from both multiplexes are included in the regression analysis and the effect of Multiplex score 1 is adjusted for the weighted effect of mutations in genes targeted in Multiplex 2 and vice versa; (3) the effect of CRISPR/Cas9-induced mutations in zebrafish orthologues of human obesity genes on obesity-related traits in offspring of CRISPR/Cas9 founders of Multiplexes 1 and 2; (4) the effect of CRISPR/Cas9-induced mutations on cardiometabolic outcomes in CRISPR/Cas9 founders targeted at orthologues of one human gene at a time; and (5) the effect of CRISPR/Cas9-induced mutations on food intake in CRISPR/Cas9 founders (AB). * effect of each additional mutated allele weighted by mutations’ predicted effect on protein function in the F_1_ generation. ** effect of frameshift and/or premature stop codon-introducing mutations in both alleles vs. no mutated alleles in the F_1_ generation. *** effects in CRISPR/Cas9-edited larvae vs. controls in the F_0_ generation.

Flow charts illustrating the experimental design are shown in Fig. 1. A summary of all main results is shown in Fig. 2.

### Overfeeding increases adiposity in zebrafish larvae

**Fig. 3:**
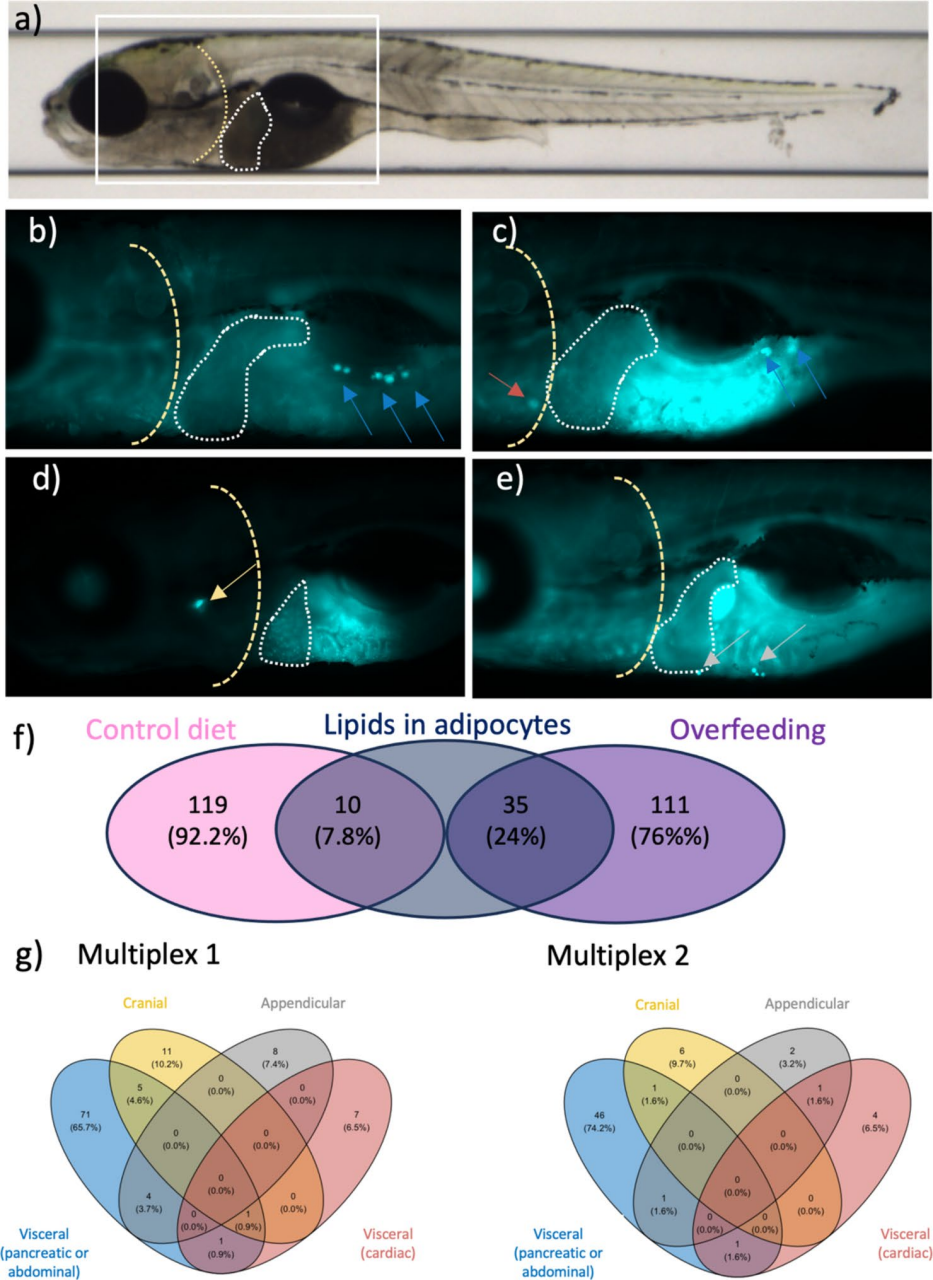
Lipid deposits observed in several anatomical regions in 10-day-old zebrafish larvae.** a)** Whole-body bright field image of a zebrafish larva in lateral orientation. The white rectangular box indicates the location of magnified images in the lower panel; the white dotted line depicts the liver area and the yellow dashed line indicates the edge of the operculum. **b**-**e)** Fluorescence microscopy images at 10x magnification of 10-day-old, Tg:2.8*fabp10a*:GFP positive zebrafish larvae illustrating the location of dye-stained lipid droplets (highlighted by arrows). The majority of the lipids were stored in the visceral region (abdominal or pancreatic, blue arrow; cardiac, red arrow), followed by the cranial region (yellow arrow), and the appendicular region (grey arrow). **f**) Results from inspection of 275 10-day-old imaged larvae raised under normal feeding conditions (*n* = 129) or overfed three-times more (*n* = 146) for the presence of lipids in adipocytes. Percentages are shown in parenthesis. **g)** In Multiplexes 1 (left) and 2 (right), a total of 108 (28%) and 62 (16%) larvae had quantifiable lipid deposits in at least one anatomical region, respectively. Most lipids were stored in the abdominal region, followed by the opercular region.

To explore if a large-scale genetic screen for obesity-related traits in 10-day-old zebrafish larvae is worthwhile, we first examined the effect of overfeeding from 5 to 10 days post-fertilization (dpf) on lipid accumulation in adipocytes, as well as on other obesity-related traits. To this end, we overfed 366 Tg(−1.2*insH2b*:mCherry; 2.8*fabp10a*:GFP) positive zebrafish larvae (AB) on regular dry food (158 imaged; 208 biochemistry), while 350 sibling controls were fed 3x less (145 imaged; 205 biochemistry, Fig. 1a). Of the overfed larvae, 24% have lipid accumulation in adipocytes at 10 dpf, compared with 7.8% in the control-fed larvae (Fig. 3f). The lipid deposits we observe are mostly located in the abdominal/pancreatic region (*n* = 31), although we also observe deposits in the appendicular (*n* = 7), cranial and cardiac regions (*n*= 4) where they were not previously observed at this early stage of development^15,22^ (Fig. 3g). Our results show that five days of overfeeding increases the odds of lipid accumulation in adipocytes (OR 4.19, 95% CI 1.95–8.98, Supp Table 1, Supp Fig. 2). Five days of overfeeding also affects developmental traits like eye diameter (beta ± SE −0.62 ± 0.18 SD units) and body length (−0.64 ± 0.19); as well as whole-body LDLc (1.02 ± 0.19), triglyceride (0.92 ± 0.23), total cholesterol (0.81 ± 0.21) and glucose (1.52 ± 0.22) content (Fig. 2, Supp Figs. 1 and 2, Supp Table 1).

## CRISPR/Cas9-induced mutations in targeted genes

We next examined the effect of mutations in 15 zebrafish orthologues of 12 established human obesity genes on cardiometabolic traits using a multiplexed CRISPR/Cas9 approach^30^. Two sets of eight (*arid5b*, *lepr*,* mc4r*, *negr1*, *pcsk1*, *pomca*, *pomcb*, *sec16b*; i.e., “Multiplex 1”, *n* = 628) and seven (*bdnf*,* irs1*, *irs2a*, *irs2b*, *sh2b1*, *sim1a*, *sim1b*; i.e., “Multiplex 2”, *n* = 585) zebrafish orthologues were targeted simultaneously, using guide RNAs with an anticipated moderate efficiency (Fig. 1b, Supp Tables 2, 3 and 4). Founders were raised and in-crossed to yield offspring with 0–2 mutated alleles in all eight and seven targeted genes. For each multiplex, 768 F_1_ larvae were overfed for five days before phenotypic characterization at 10 dpf (Fig. 1b, Supp Fig. 3), and paired-end sequencing at the CRISPR/Cas8-targeted sites.

Across the eight and seven targeted sites (Supp Table 3), we observe 242 unique alleles in the 2 × 768 characterized F_1_ larvae, ranging from three (*bdnf* and *sim1a*) to 42 (*mc4r*) unique alleles per targeted site (Supp Table 5). Most CRISPR/Cas9-induced DNA breaks result in frameshift (46.1%) or missense mutations (29.8%, Supp Fig. 4, Supp Table 6). The number of mutated alleles is normally distributed across all targeted sites (Supp Fig. 5), with mutant allele frequencies (MAFs) ranging from 2.7% for *pomcb*– a presumed pseudogene^32^ – to 86.1% for *sim1b* (Supp Table 7). We generally observe higher MAFs for genes targeted in Multiplex 2 (Supp Fig. 6), and for single gRNAs with higher efficiency in a priori performed test injections (Supp Figs. 6 and 7; Supp Tables 3 and 7), although the predicted efficiency in test injections correlates poorly with the MAF in either founders or F_1_ larvae (Supp Fig. 7).

Within each zebrafish orthologue, conclusions are preferentially drawn from a phenotypic comparison of larvae with both alleles affected by frameshift and/or premature stop codon introducing mutations (FS/PS mutations) vs. larvae with no affected alleles (homozygous-like model, 2 vs. 0 mutated alleles). For image-based traits, this comparison is feasible for *mc4r* (22 vs. 171), *negr1* (54 vs. 105), *pcsk1* (12 vs. 177), *sec16b* (20 vs. 155), *irs2b* (16 vs. 141), *sh2b1* (42 vs. 57), and *sim1b* (268 vs. 24, Supp Table 7). For orthologues with < 10 larvae affected by FS/PS mutations in both alleles or with < 10 larvae free from CRISPR/Cas9-induced mutations, an additive model is used instead. For the additive model, we used continuous variables as exposures containing information on whether larvae carried 0, 1 or 2 mutated alleles, weighted by the predicted effect of variants on protein function using Variant Effect Predictor (see methods). For human genes with two zebrafish orthologues (i.e., *POMC*, *SIM1*, and *IRS2*), a comparison of larvae with FS/PS mutations in all four alleles vs. larvae without mutations in any allele is not feasible using the data (Supp Table 7). To increase the statistical power to find genetic effects, we pooled data across both multiplexes in the analysis, thereby increasing the number of larvae without mutated alleles for all genes.

## Genetic burden scores are not associated with obesity-related traits

**Fig. 4:**
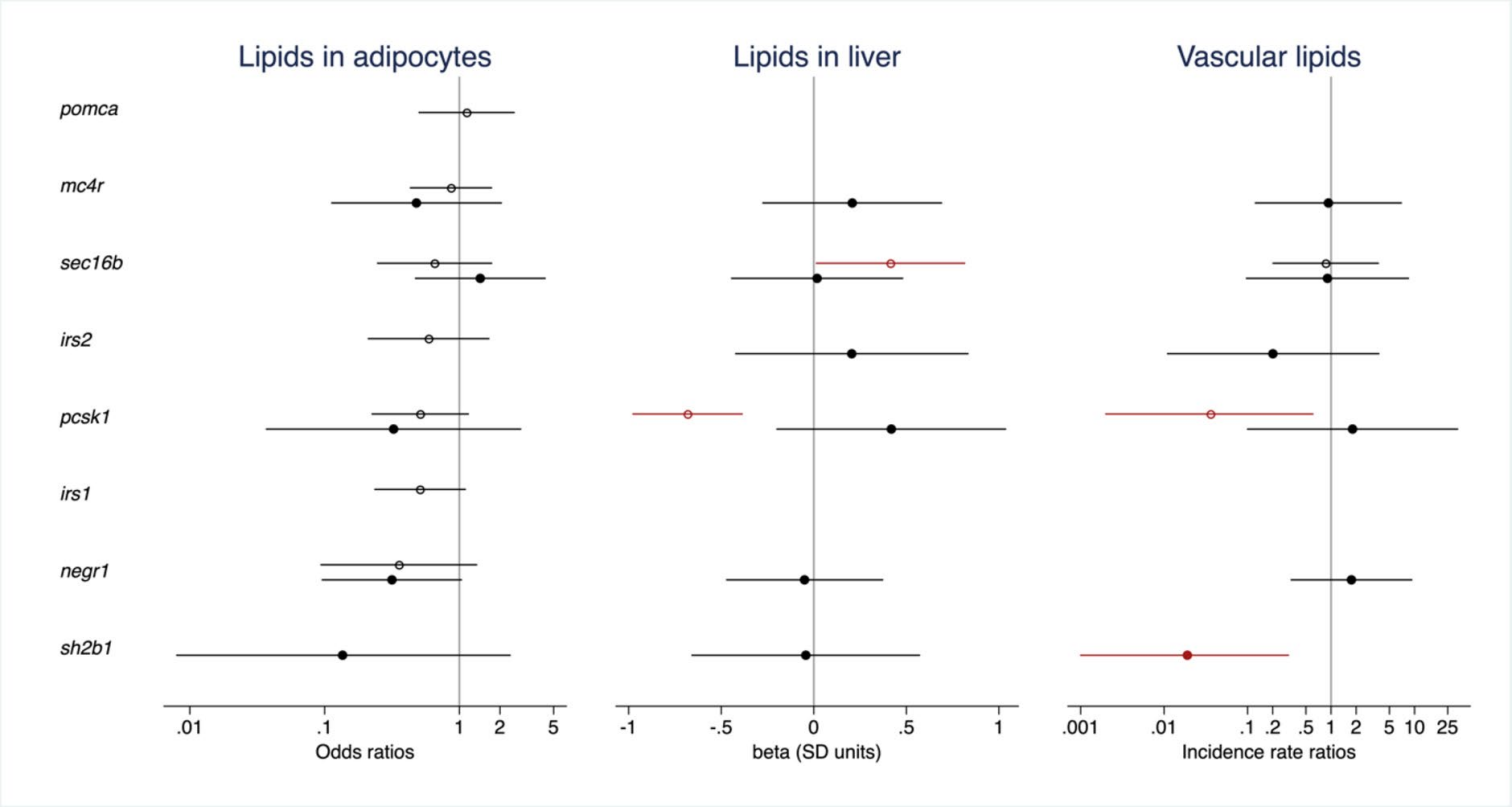
The effect of CRISPR/Cas9-induced mutations on lipid deposition in adipocytes and ectopic regions in 10-day-old zebrafish larvae. Results are shown for genes with data from (1) a comparison of CRISPR/Cas9 edited founders (F0 generation) for all zebrafish orthologues of a human gene vs. sibling controls (open circles) and/or (2) from a comparison of offspring from an in-cross of CRISPR/Cas9 founders (F_1_ generation) with frameshift and/or premature stop codon introducing mutations vs. larvae free from CRISPR/Cas9-induced mutations in the gene (filled circles). Dots and error bars show odds ratios (logistic regression, for lipid deposition in adipocytes, left), effect sizes expressed in standard deviation units (linear regression, for hepatic lipid deposition, middle), and incident rate ratios (negative binomial regression, for vascular lipid deposition, right) and their 95% confidence intervals. In the F_0_ experiment, effects are adjusted for batch, tank and time of day at imaging. In the F_1_ experiment, effects are adjusted for multiplex (1 or 2), batch, time of day at imaging, and the number of mutated alleles in the remaining CRISPR/Cas9-targeted genes weighted by the predicted effect of such mutations on protein function (synonymous 0.33, missense 0.66, frameshift and/or premature stop codon introducing 1.00). Effects with *P* < 0.05 are shown in red. Of note: F_1_ results for *irs2* (filled circles) are for *irs2b* only, since the data does not facilitate a comparison of larvae with frameshift and/or premature stop codon introducing mutations in both *irs2a* and *irs2b* vs. controls.

Across Multiplexes 1 and 2, we observe lipid accumulation in adipocytes in 22% of the 2 × 384 imaged larvae, mostly in the abdominal visceral region (Fig. 3, Supp Table 8). We first examined the association of obesity-related traits with two genetic burden scores, each comprising the number of mutated alleles across all CRISPR/Cas9-targeted sites weighted by the predicted effect of mutations on protein function^33^ (Supp Fig. 8). Unexpectedly, odds of lipid accumulation in adipocytes are lower in larvae with a higher genetic burden across all targeted sites in Multiplex 1 (score 1, OR 0.85, 95% CI 1.84–8.14, *p* = 0.037, Figs. 2 and 4; Supp Fig. 9; Supp Table 9 and 10), possibly reflecting the high heterozygosity rate at CRISPR/Cas9-targeted sites in Multiplex 1. Interestingly, body length is not affected by the high heterozygosity rate in Multiplex 1 or 2 (*p* = 0.804 and *p* = 0.117, respectively, Supp Fig. 9, Supp Table 10). In line with this, the effect of Multiplex scores 1 and 2 on lipid accumulation in adipocytes is unaffected by adjusting for body length (Supp Table 10). Glucose content is positively associated with the genetic burden score for Multiplex 2 (score 2, 0.07 ± 0.03, Figs. 2 and 4; Supp Fig. 9; Supp Table 9 and 10).

## Effects of mutations in autosomal recessive monogenic obesity genes

We next examined the effect of mutations in individual genes on image- and biochemistry-based cardiometabolic outcomes, starting with the three autosomal recessive monogenic obesity genes: *PCSK1*,* LEPR*, and *POMC*.

Larvae with FS/PS mutations in both *pcsk1* alleles have a smaller dorsal (−0.57 ± 0.24) and lateral (−0.61 ± 0.24) body area normalized for length than larvae free from CRISPR/Cas9-induced mutations (Fig. 2; Supp Figs. 9, 10, 11, 12 and 13; Supp Table 11). These results are inconsistent with an elevated risk of obesity in PCSK1-deficient patients^7^, but consistent with delayed growth in *Pcsk1*-null mice^34^. Compared with larvae free from CRISPR/Cas9-induced mutations in *pcsk1*, zebrafish larvae with FS/PS mutations in both *pcsk1* alleles have fewer beta cells and a smaller pancreatic islet, but a similar mean beta cell insulin expression and similar whole-body glucose levels (Supp Fig. 13, Supp Table 11).

Results for the effect of mutations in *lepr* and *pomca* should be interpreted with caution, due to the low number of larvae with FS/PS mutations in both alleles (i.e., 10 and 18 respectively; Supp Figs. 4 and 10, 11, 12 and 13; Supp Table 7). While impairments in *LEPR *signaling increase susceptibility to early-onset obesity, hyperphagia, insulin resistance, and infertility in humans^3^, *db*/*db *mice^35^, and *fa/fa *rats^36^, we do not observe effects of mutations in *lepr* on any adiposity-related trait in 10-day-old overfed zebrafish larvae (Fig. 2, Supp Fig. 13, Supp Table 10). This is consistent with previously reported results from juvenile *lepr*^−/−^zebrafish by 40 dpf^37^, and may reflect the low sequence homology across zebrafish *lepr* and human *LEPR* (i.e., 27%, Supp Table 3).

Each additional mutated allele in *pomca* results in a larger lateral and dorsal body area normalized for body length – analogue to a higher BMI – and in a lower glucose content (Supp Fig. 13, Supp Table 10). In line with this, patients with POMC deficiency present with severe early-onset obesity and hyperphagia^6^, and *Pomc*-null mice are obese from around 2 months of age, but have normal glucose tolerance^38^. An earlier study in zebrafish larvae also did not observe differences in beta cell traits or whole-body triglyceride or cholesterol levels at 5 dpf, but showed enhanced somatic growth by 90 dpf, without affecting glucose levels^32^.

In summary, results for mutations in *pcsk1* and *lepr* on adiposity-related traits are discordant with observations in mammalian models, while the role of *pomca* seems conserved from zebrafish to mammals.

## Effects of mutations in dominant obesity genes

We next examined the effect of mutations in zebrafish orthologues of the dominant obesity genes *SH2B1*, *SIM1*, *BDNF*, and *MC4R*. Compared with larvae free from mutations in *sh2b1*, larvae with FS/PS mutations in both *sh2b1* alleles have lower whole-body levels of LDLc and less vascular lipid deposition (Supp Fig. 13; Fig. 4; Supp Table 11). SH2B1 mediates cell signaling responses in both central and peripheral tissues. Individuals with heterozygous mutations in *SH2B1*(e.g., those encoding P90H, T175N, P322S, and F344LfsX20) have been diagnosed with obesity, insulin resistance and type-2 diabetes^39^. In mice, genetic disruption of *Sh2b1* also results in obesity, insulin resistance, type-2 diabetes, severe leptin resistance, and metabolic dysfunction-associated steatotic liver disease. Mice with targeted hepatic deletion of *Sh2b1 *fed on a high-fat diet present with normal body mass and glucose metabolism and have lower hepatic VLDL secretion and attenuated hepatic steatosis. This suggests Sh2b1 regulates hepatic triacylglycerol synthesis, lipolysis and VLDL secretion^40^. In line with this, we observe lower LDLc levels in zebrafish larvae with mutations in s*h2b1*, which may contribute to their lower levels of vascular lipid deposition.

In humans, chromosomal aberrations in *SIM1 *lead to severe, early-onset hyperphagic obesity^41^. While hyperphagia, obesity, and hyperinsulinemia are also observed in *Sim1 *haploinsufficient mice^42^, we do not observe obesity-related phenotypes in zebrafish larvae. No larvae carry FS/PS mutations in all four *sim1a* and *sim1b* alleles. FS/PS mutations in both *sim1b* alleles result in more vascular lipid deposition (Fig. 2; Supp Fig. 13; Supp Table 11).

No characterized larvae have FS/PS mutations in both *bdnf* alleles. Compared with larvae free from mutations in *bdnf*, the 56 larvae carrying one mutated *bdnf* allele have more hepatic lipid accumulation (Fig. 2; Supp Fig. 13; Supp Table 10). In absence of adequate statistical power to detect an effect on whole-body adiposity, elevated lipid accumulation in the liver of larvae with one remaining *bdnf* allele could be considered directionally consistent with the obese phenotype observed in *BDNF *haploinsufficient humans^43^, as well as with diabetic *db*/*db *mice showing less hepatic steatosis upon subcutaneous injection with Bdnf^44^.

In spite of an adequate MAF (i.e., 41.6%), we do not observe an effect of mutations in *mc4r* (Figs. 2 and 4; Supp Fig. 13; Supp Tables 7, 10 and 11) on any outcome. Previous studies in zebrafish showed effects of *mc4r *mutations on higher food intake in post-juvenile stages, as well as on elevated body mass, body fat percentage, and insulin resistance in adult fish^45^. Hence, it seems likely that 10 dpf is too early to detect a role for *mc4r* in energy balance in zebrafish larvae.

In summary: mutations in *sh2b1* and *bdnf* result in directionally consistent results across species, while the inconsistent effects observed across species for mutations in *SIM1* and *MC4R* orthologues may reflect compensation by an intact allele, and the early developmental stage at phenotyping, respectively.

### Effect of mutations in putative causal genes for polygenic obesity

Finally, we examined the effect of mutations in zebrafish orthologues of several genes associated with polygenic forms of obesity that previously showed some evidence of a causal role (i.e., *NEGR1*, *SEC16B*, *ARID5B*, *IRS1*, and *IRS2)*. Of all perturbed genes, only mutations in *negr1* tend to influence lipid accumulation in adipocytes (−1.15 ± 0.61; *p* = 0.056, Figs. 2 and 4; Supp Table 11; Supp Fig. 13). The identification of two 43 and 8 kb deletions upstream of *NEGR1* were amongst the first common variants to be associated with the regulation of BMI, implicating *NEGR1 *in the etiology of obesity^46,47^. The 8 kb deletion removes the binding site for transcriptional repressor NKX6.1, leading to a higher *Negr1* expression and lower odds of obesity. Functional studies in mice showed that knockdown of *Negr1 *reduces body mass; decreases food intake and locomotor activity^48^; and increases fat mass, with abnormally enlarged adipocytes and abnormal lipid deposition in the liver^49^. Hence, the effects we observe in zebrafish larvae are inconsistent with effects observed in humans and mice.

While mechanistic studies previously implicated *SEC16B*^50^ and *ARID5B*^51^ as effector genes in GWAS-identified loci for obesity, we do not detect effects of CRISPR/Cas9-induced mutations on obesity-related traits (Figs. 2 and 4, Supp Fig. 13, Supp Tables 7, 10 and 11).

As mentioned earlier, no larvae in Multiplex 2 are free from mutations in *irs1* and *irs2a*, so the effect of mutations in these genes can only be examined in comparison with larvae from Multiplex 1 (which typically carry CRISPR-induced mutations in other candidate genes, Supp Fig. 5). In mice, disruption of *Irs1 *leads to impaired growth^52^, glucose intolerance, hyperinsulinemia, and insulin resistance after an insulin challenge, despite being lean^53^. The lean phenotype is caused by the inability of embryonic fibroblasts to differentiate into white adipose tissue in *Irs1* and *Irs2 *knockout mice^54^. Our results show that each additional mutated *irs1 *allele results in shorter and leaner larvae, albeit, without the effects on glucose or insulin metabolism observed in mice^54^ (Fig. 2; Supp Fig. 13; Supp Table 10).

**Fig. 5:**
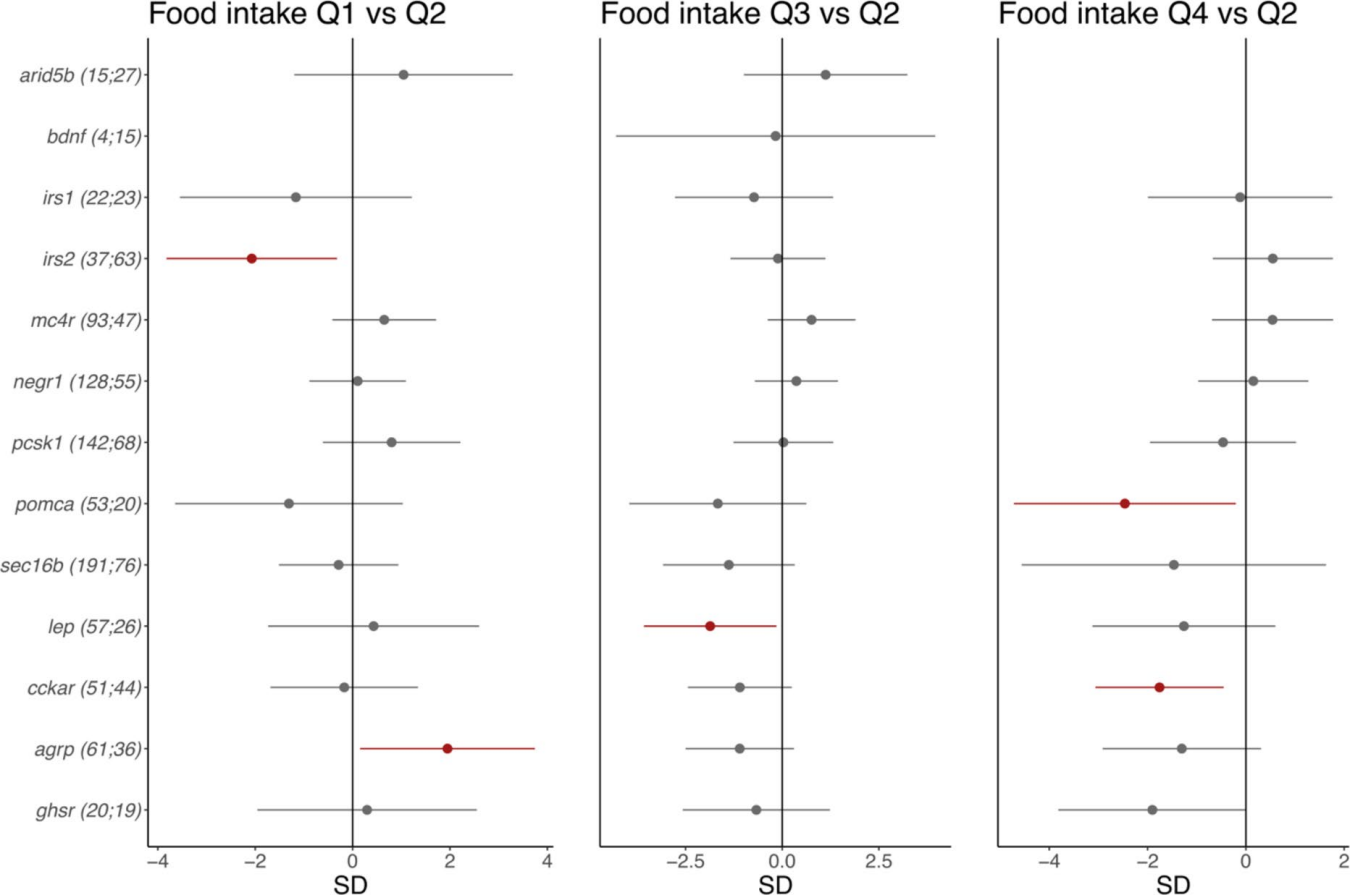
The effect of CRISPR/Cas9-induced mutations in 13 obesity genes on food intake in 8-day-old CRISPR/Cas9 founders. Dots and error bars show effect sizes and 95% confidence intervals for affected larvae (number on the left) vs. controls (number on the right). The effect of being in the first quartile of food intake [Q1]; left); the effect of being in the third quartile (Q3; middle); the effect of being in the top quartile (Q4; right). All are compared with the second quartile (Q2) as a reference. The results for *irs2* and *lep* are derived from the concurrent targeting of both orthologues (i.e., *irs2a* and *irs2b*; *lepa* and *lepb*). Effects have been adjusted for batch, tank and time of day at imaging. Effects with *P* < 0.05 are shown in red.

We do not observe an effect of mutations in *irs2a*; while larvae with FS/PS mutations in both *irs2b* alleles have higher whole-body LDLc and total cholesterol levels (Supp Fig. 13, Supp Table 11). Obese individuals and people suffering from metabolic dysfunction-associated steatotic liver disease on average have lower levels of hepatic IRS2 ^9^. Disrupting *Irs2 *in mice results in higher triglyceride content in adipocytes, hyperphagia due to higher agouti-related peptide, and lower hypothalamic POMC expression, higher body weight^55^, and progressive loss of beta cell function^56^.

In summary, we observe directionally consistent effects of mutations in orthologues of *irs1* on body size parameters compared with humans and/or mice. We detect directionally inconsistent results across species for mutations in *negr1*, and no effect in zebrafish larvae for mutations in *sec16b* and *arid5b*. However, results for *IRS1*, *IRS2* and *ARID5B* orthologues should be interpreted with caution due to suboptimal MAFs.

## Effects of CRISPR/Cas9-induced mutations on obesity traits in founders

The very low or very high MAFs we observe for some genes in the offspring of multiplexed CRISPR/Cas9 founders limits our ability to draw firm conclusions on the role of these genes in obesity-related traits in 10-day-old zebrafish larvae. Moreover, for genes targeted in multiplex, we cannot rule out effects on viability prior to reaching sexual maturity – or on fertility or fecundity – either per se, or due to a high genetic burden when targeted alongside other genes. Therefore, we next examined the effect of mutations in these genes one human gene at a time, straight in 10–11 day-old, overfed, transgene positive founders (for *mc4r*, *pomca*, *irs1*, *irs2a + irs2b*, *negr1*, *pcsk1* and *sec16b*) targeted using a more efficient CRISPR/Cas9 approach^31^ (Figs. 1c and 4, Supp Tables 12 and 13). This resulted in 100 to 180 affected larvae and 17 to 81 sibling controls per human gene, with lipid accumulation in adipocytes observed in 22.5 ± 6.8% of larvae. Despite the much-improved statistical power to detect genetic effects compared with the F_1_ experiment, we observed no significant effects of CRISPR/Cas9-induced mutations in any gene on lipid accumulation in adipocytes in the F_0_ study either (Fig. 4, Supp Tables 14 and 15). Noteworthy though are non-significant lower odds in larvae with mutations in *negr1* (*P* = 0.12, directionally consistent with results in the F_1_ generation) and *irs1* (*P* = 0.088, directionally consistent with humans, Fig. 4).

To examine differences between multiplexed F_1_ larvae and F_0_ larvae, we selected two genes for which we anticipate to have adequate statistical power to find effects of mutations in the F_1_ experiment (i.e., *pcsk1* and *sec16b*) and studied the effect of CRISPR/Cas9-induced mutations on other cardiometabolic traits (Figs. 2 and 4, Supp Tables 16 and 17). Just like in multiplexed F_1_ larvae, CRISPR/Cas9 founders with mutations in *pcsk1* (*n* = 138) have a smaller dorsal and lateral body area normalized for length than their sibling controls (*n* = 67). Moreover, CRISPR/Cas9 founders with mutations in *pcsk1* are shorter than sibling controls (−0.76 ± 0.12, Supp Fig. 14, Supp Table 14), consistent with stunted linear growth in *PCSK1*-deficient children^7^. However, in contrast with the F_1_ experiment, F_0_ larvae affected at *pcsk1* have a similar beta cell mass, a smaller liver; lower whole-body LDLc, triglyceride, total cholesterol and glucose content; and less hepatic and vascular lipid deposition than sibling controls (Fig. 4, Supp Fig. 15, Supp Table 16). A lower LDLc^57^and glucose^7^ content in larvae with CRISPR/Cas9-induced mutations in *pcsk1* is consistent with previously described results in humans and murine models.

We also compared F_0_ larvae with CRISPR/Cas9-induced mutations in *sec16b* (*n* = 191) with sibling controls (*n* = 76) and show that affected larvae are longer and have more lipid accumulation in the liver, which despite not affecting lipid accumulation in adipocytes may reflect higher odds of obesity (Figs. 2 and 4; Supp Figs. 14 and 15; Supp Tables 14 and 17). No effects are observed for this gene in the multiplexed F_1_ larvae, likely reflecting the difference in sample size. Intestine-specific *Sec16b *knockout mice were recently shown to have impaired chylomicron lipidation and protection from high-fat diet-induced obesity and glucose intolerance^50^. With SEC16B expressed in intestine and liver, a comparison between globally targeted zebrafish larvae and tissue-specific knockout mice is not straightforward.

## Effect of mutations on food intake

Several of the 12 human genes we studied influence food intake regulation in mammals, in some cases starting from birth (e.g., *PCSK1*, *MC4R*, *LEPR*, *POMC*, *SIM1*). While 10 dpf may be (too) early to detect potentially small early effects on lipid accumulation in adipocytes, we next examined if mutations in these and four additional genes (that is, leptin -*lep*-, cholecystokinin A receptor -*cckar*-, agouti-related neuropeptide -*agrp*-, ghrelin receptor -*ghsr*-) affect food intake in zebrafish larvae. All zebrafish orthologues of a human obesity gene were targeted together and the effect on food intake was estimated by comparing the amount of ingested, fluorescently labeled food in 8 dpf larvae with CRISPR/Cas9-induced mutations and their sibling controls (Fig. 1c; Supp Fig. 16; Supp Tables 12 and 18). We divided the food intake variable into quartiles and used the second quartile (Q2) as a reference for food intake comparisons between affected larvae and controls (Fig. 5).

Experiments show directionally anticipated results for two genes. First, larvae with CRISPR/Cas9-induced mutations in *irs2a* and *irs2b* (*n* = 37) are at lower risk of being in the first quartile of food intake (Q1) vs. the second quartile (Q2) than sibling controls (*n* = 63) (Figs. 2 and 5; Supp Tables 18 and 19). This result is directionally consistent with *Irs2 *knockout mice eating more^55^, and with established effects of mutations in *IRS2 *on obesity^9^. Secondly, larvae with CRISPR/Cas9-induced mutations in *agrp* (*n* = 59) are at higher risk of being in Q1 vs. Q2 than sibling controls (*n* = 34), in line with agouti-related protein’s anticipated orexigenic activity, as well as with overexpression of *agrp *in adult zebrafish resulting in obesity, increased growth and adipocyte hypertrophy^58^. For a third gene (*ghsr*), mutations may have a directionally anticipated effect on food intake in zebrafish larvae, but results do not reach significance. In spite of being expressed in endocrine pancreatic cells in adult zebrafish, ghrelin receptor may thus play a similar role in food intake regulation in zebrafish larvae as compared with its mammalian orthologue^59^.

Larvae with CRISPR/Cas9-induced mutations in *pomca* (*n* = 53) are at lower risk of being in the top quartile of food intake (Q4) vs. Q2 than sibling controls (*n *= 20). This result is directionally consistent with a previous study in 7-day-old zebrafish larvae^60^, but at odds with observations in mammalian models and human patients^6^. Also, larvae with CRISPR/Cas9-induced mutations in *lep* (*n* = 55) are at lower risk of being in the third quartile (Q3) vs. Q2 than sibling controls (*n*= 25). This is in line with earlier observations in adult zebrafish, where leptin seems to play a role in stress regulation, osmoregulation^61^, and glucose homeostasis^62^, rather than in regulating food intake and energy balance^62^. However, it is directionally inconsistent with observations in humans^63^, *ob*/*ob *mice^35^, and *fa/fa *rats^36^. Finally, larvae with CRISPR/Cas9-induced mutations in *cckar* (*n* = 51) are at lower risk of being in the top quartile (Q4) vs. Q2 compared with controls (*n*= 44). This contrasts with fasting-to-refeeding studies in adult fish – in which cckar acts as an appetite-suppressing factor^58^– as well as with results from a variety of vertebrate species, where activation of CCKAR by cholecystokinin – a gastrointestinal hormone that regulates appetite – reduces food intake^64^.

We do not observe effects on food intake for CRISPR/Cas9-induced mutations in *arid5b*, *mc4r*, *negr1*, *pcsk1*, *sec16b*; and *irs1* (Figs. 2 and 5; Supp Tables 18 and 19). Low survival in larvae with CRISPR/Cas9-induced mutations prevents us from drawing conclusions about the role in food intake for mutations in *lepr*, *sh2b1*, *bdnf*, and *sim1a + sim1b* (Supp Table 20).

In summary, of the 16 human genes with a known effect on obesity, we observe effects on food intake for orthologues of five genes. For two human genes (i.e., *irs2a + irs2b* and *agpr*), the effect of mutations on food intake is directionally consistent across zebrafish larvae and mammals. For three others genes (*lep*, *pomca*, *cckar*), the direction of effect is inconsistent across zebrafish and mammals, but consistent with earlier findings in zebrafish for *lep* and *pomca*).

## Discussion

Here we explored the merit of an image- and CRISPR/Cas9-based pipeline to systematically characterize the role of genes in obesity-related traits using 10-day-old zebrafish larvae. We aimed to predispose larvae to obesity by overfeeding them from 5 to 10 dpf, a method thus far mainly used to study the probability of accumulating lipids in adipocytes in adult zebrafish^27,28^. Research in larvae was previously performed in < 100 larvae per study, and with larvae fed on a high-fat diet^65^. While adipose tissue could be clearly visualized across several anatomical regions in adult fish^15^, adipose tissue development typically starts around 8 dpf in zebrafish larvae, with adipocytes supposedly detectable from 12 dpf or a body length of ~ 4.3 mm onwards (Supp Fig. 17)^22^. Adipocytes can be visualized and quantified in larvae by incubation with fluorescent dyes^25^. In previous studies, lipid-loaded adipocytes typically localize abdominally or close to the pancreas^15,22^. Here, 35 (24%) 10 dpf overfed larvae show lipid accumulation in adipocytes. In Multiplexes 1 and 2, 108 (28%) and 62 (16%) larvae show lipid deposits in adipocytes, compared with 36 (24%) overfed larvae free from CRISPR/Cas9-induced mutations in the overfeeding experiment. While 86% of lipids in adipocytes are in the abdominal or pancreatic region, we also observed the presence of lipid depots in regions where they had not previously been observed at this early stage of development, such as in the cranial, appendicular and cardiac regions. In spite of the low proportion of larvae with lipid deposition in adipocytes at 10 dpf, we observed a positive effect of overfeeding, suggesting that a genetic screen in overfed larvae is worthwhile. However, our multiplexed F_1_ screen only yielded an adequate number of mutants and controls for five of the twelve human genes (i.e., *MC4R*, *NEGR1*, *PCSK1*, *SEC16B*, *SH2B1*). In combination with the low proportion of larvae with lipid accumulation in adipocytes (22%), this meant we had low statistical power to detect true genetic effects on lipid accumulation in adipocytes. Aiming to overcome the low statistical power, we performed a follow-up experiment, in which we characterized the effect of mutations in *mc4r*,* pomca*,* negr1*,* irs1*,* irs2a + irs2b*,* pcsk1* and *sec16b* on lipid accumulation in adipocytes straight in a larger number of CRISPR/Cas9 founders and sibling controls. Despite a much larger sample size, we did not observe an effect of CRISPR/Cas9-induced mutations on lipid accumulation in adipocytes in the founders either, suggesting 10–11 dpf may simply be too early in development to see such effects.

Although 10–11 dpf seems too early to characterize genes for a role in lipid accumulation in adipocytes under overfeeding conditions, we show that mutations in most examined genes already influence cholesterol metabolism, glucose metabolism and/or ectopic lipid accumulation by this stage of development. This implies that systematically characterizing the role of obesity candidate genes using this pipeline could improve our understanding of their role in cardiometabolic traits through pathways that are independent of excess adipose tissue. Furthermore, the small number of differentiated adipocytes at this developmental stage could model mechanisms that resemble a state in obese humans where adipose tissue fails to expand and lipids start accumulating in ectopic tissues instead (e.g., in liver, skeletal muscle or vasculature), affecting peripheral insulin sensitivity. Our results, together with those of other studies, show image-based analyses in 10-day-old zebrafish larvae are useful to visualize genetic effects on traits related to type-2 diabetes^66^, steatotic liver disease^67^and coronary artery disease^69^.

Genetic effects on food intake can already be detected at 8 dpf, with effects in the anticipated direction for mutations in orthologues of *AGRP* and *IRS2*, and possibly *GHSR*. However, inconsistent directions of effect on food intake across zebrafish larvae and mammals for mutations in orthologues of *LEP*, *CCKAR*, and *POMC*in our and other studies^32,61,62^ suggests that systematic screens for genetic regulators of food intake in zebrafish larvae are not an obvious next step when pursuing results that are relevant for humans.

In this study, we used both a multiplexed approach, in which we characterized stable mutants for up to eight genes simultaneously in the F_1_ generation^30^, as well as an approach in which we characterized larvae for mutations in all orthologues of one human gene at a time, straight in the CRISPR/Cas9 founders (i.e., the F_0_ generation), using a more efficient CRISPR/Cas9 approach^31^. Both approaches have strengths and weaknesses. The multiplexed approach allows characterizing the role of orthologues of multiple human genes simultaneously. By characterizing larvae in the F_1_ generation, all larvae have stable genotypes, and paired-end sequencing of the region around the CRISPR/Cas9 cut site allows quantification of the exact CRISPR/Cas9-induced mutation(s) each larva carries at each of the targeted sites. This information can be used to predict – for each allele and at each targeted site and larva – the effects of mutations on protein function. A disadvantage of the multiplexed approach is that we aim for moderate targeting efficiency in the founders, to ensure the F_1_ generation contains larvae with 0, 1 and 2 mutated alleles at each targeted site. Larvae with a higher overall genetic burden across all simultaneously targeted sites are also more likely to have lower survival, fertility and fecundity. Since sequencing is performed after imaging, there is a risk that the statistical power is low due to very low – or very high – mutant allele frequencies in the offspring. This is especially prudent for human genes with > 1 orthologue, where the number of F_1_ larvae in which all alleles of all orthologues are mutated is by definition low. Due to suboptimal mutation rates, we can only draw robust conclusions about the role of five of 12 human genes examined in the F_1_ screen. In addition, the role of genes is inferred by comparing larvae with mutations in said gene with larvae free from mutations in that gene, most of which still carry mutations at the other targeted sites in the multiplexed approach. While we adjust effects of mutations in gene x for the effect of mutations in all other targeted genes, this at best reduces the power to detect true effects. In the presence of epistasis, adjusting is not sufficient, and may result in biased estimates, since the statistical power is too low to systematically quantify the presence or absence of such gene-gene interactions. Here, we targeted several genes known to act in the leptin-melanocortin pathway together in Multiplex 1 (i.e., *lepr*, *mc4r*, *pcsk1*, *pomca* and *pomcb*), which may have influenced the results. Finally, the multiplexed F_1_ approach requires an extra generation before being able to characterize the offspring.

Many of the limitations of the multiplexed approach are addressed in the F_0_ approach, while retaining strengths like semi-automated image acquisition across several tissues simultaneously in the same animals^68–70^; and deep learning-based image segmentation. The F_0_ approach is based on the assumption that targeting a gene with highly mutagenic duplex gRNAs – possibly even at several locations per gene – results in a high proportion of larvae with biallelic mutations^31^. By targeting orthologues one human gene at a time, the risk of epistasis is reduced, while the statistical power is increased by removing the need to adjust for the effect of CRISPR/Cas9-induced mutations in other targeted genes. The F_0_ approach also has the advantage of reducing the time required to complete the screen, and of identifying issues like reduced viability of affected larvae sooner than in the multiplexed approach. In addition, the sample size is only limited by the number of injected eggs and larval survival. The F_0_ approach also presents some limitations. Experiments are performed in mosaic F_0_ larvae, and therefore not all cells in the tissue of action are guaranteed to be successfully targeted by CRISPR/Cas9. Since duplex gRNAs are designed to preferentially introduce frameshift mutations, injections are performed at the single-cell stage, and the duplex RNA approach is said to cleave the DNA at an earlier stage in cell division than the older multiplexed approach^31^, the number of affected tissues is maximized. Lastly, we acknowledge that since we examined genetic effects on the probability of accumulating lipids in adipocytes at day 10, we cannot rule out that other factors like growth rate and developmental stage have influenced the results. For this reason, we provide results from a secondary statistical model in which we additionally adjust for body length. However, these results should be interpreted with caution, since mutations in some genes (like *PCSK1*) are known to affect linear growth, so adjusting for length could introduce bias. Finally, we did not examine developmental stage of the larvae and, given the possibility of decoupled development and growth^71^, developmental stage may influence the phenotype of interest.

Here, we obtain a 2–8-fold larger number of affected larvae in the F_0_ screen compared with the number of larvae with FS/PS mutations in both alleles in the F_1_ screen. As a result, we observed some effects in the F_0_ screen only, above and beyond the effects observed in both screens. All the above-mentioned advantages, such as a larger effective sample size, higher statistical power, and reduced screening time, make the F_0_ screen the preferred approach moving forward.

In conclusion, while our pipeline can be used to efficiently characterize effects of mutations in candidate genes on a range of obesity-related cardiometabolic traits in each and every larva, 10 and 11 dpf seems too early in development to efficiently detect effects on lipid accumulation in adipocytes for genes with a conserved role between species. Moreover, for some genes that play a role in the leptin-melanocortin pathway, we did not detect an effect on traits in the expected direction, suggesting that the role of the pathway in food intake regulation may not be conserved between mammals and fish, or that effects only become apparent during later stages of development. We observed effects on food intake in the expected direction for mutations in orthologues of genes that are not directly involved in the leptin-melanocortin pathway (i.e., *IRS2*, *AGRP*). Furthermore, we detected more robust cardiometabolic effects in the F_0_ screen than in the F_1_ screen, suggesting that our phenotypic screen allows detection of the role of genes in several metabolic pathways even before the onset of obesity. Therefore, it will probably still proof worthwhile to systematically characterize the role of obesity candidate genes in cardiometabolic traits in 10-day-old zebrafish larvae.

## Methods

### Animal care

All experiments were performed in 8- to 11-day-old zebrafish larvae that were offspring of wildtype fish (AB) or of AB fish carrying two fluorescently labeled transgenes: Tg:−1.2*insH2b*:mCherry to label insulin expressing nuclei^72^ and Tg:2.*8fabp10a*:GFP to label the liver^73^. Adult zebrafish used to generate offspring for experiments were maintained at 28 °C in re-circulating, filtered water (Aquaneering, San Diego, CA), on a regimen of 14 h light: 10 h darkness. Adult fish were fed twice each day with dry food (Sparos, Olhao, Portugal) and rotifers. All zebrafish used in this study were purposely bred and maintained in the Uppsala University Zebrafish Facility.

### Dietary intervention

The experimental workflow is shown in Fig. 1a.

We first examined the effect of overfeeding on obesity-related traits. To this end, transgenic larvae were raised in 1 L tanks from day 5 to 9 and fed twice per day on standard dry food. The diet consisted of Golden Pearls (50–100 μm particles, Alcester, UK) provided at 5 mg/feeding/30 larvae (control feeding) or 3x as much (overfeeding); or of Zebrafeed (< 100 μm, Sparos, Portugal) provided iso-energetically, at 5.6 mg/feeding/30 larvae or 3x as much (Fig. 1a).

At day 10, larvae that were negative for one or both fluorescently labeled transgenes (*n* = 413) were collected for biochemistry-based analyses (see, “*Quantifying LDLc*,* triglycerides*,* total cholesterol*,* glucose and protein levels*”). Larvae positive for both fluorescently labeled transgenes (*n *= 303) were incubated in monodansylpentane (MDH) to stain neutral lipids^26^ and were used for semi-automated imaging (see “*High-throughput in vivo image acquisition for obesity-related traits*”) before being used for biochemistry-based analyses.

At the time of imaging, tanks were blinded and six larvae from each condition were stained and imaged in an alternating manner. The experiment was conducted across two days to reach the desired sample size. Effects of overfeeding were adjusted for age in days, time of day at imaging, and food brand, after having ascertained that there was no interaction with food brand for any exposure. In the remainder of the experiments, all larvae were overfed on the amounts described here.

### Genetic screening

The experimental workflow is shown in Fig. 1b-d.

### Multiplexed approach

#### Design and efficiency testing of CRISPR/Cas9 single guide RNAs

We selected 12 human genes known to play a role in obesity, which together have 15 orthologues in zebrafish (Supp Tables 2 and 3). We designed single guide RNAs (gRNAs) using ChopChop v.2.0 ^74^and CRISPRscan^75^, to induce a loss of function mutation in the targeted genes. Genes were targeted at an early exon in sequences that – within each gene – were shared across all transcripts and that were free from predicted off-target activity (Supp Fig. 10, Supp Tables 2 and 3). The single gRNAs were assembled as described by Varshney et al.^30^ and in vitro transcribed according to the manufacturer’s instructions, i.e., using a TranscriptAid T7 or MEGAscript SP6 high yield transcription kit (both Thermo Fisher Scientific, Waltham, USA). Samples were purified using the RNA Clean & Concentration-5 kit (Zymo Research, Seattle, USA) and diluted to a final concentration of 300 ng/µL. To pre-test each single gRNA’s efficiency, we microinjected 2 nL pools into fertilized eggs at the single cell stage. Each pool consisted of 250 pg single gRNA per targeted site and 300 pg of SpCas9 mRNA per pool in 0.5% Phenol Red solution (Sigma-Aldrich, Munich, Germany), to visualize successful micro-injections. Injected eggs were subsequently incubated at 28.5 °C until the morning of day 5 post-fertilization. Up to 60 un-injected embryos were reared in separate dishes, to monitor mutation-driven effects on mortality and/or gross anatomical deformations during early development. At 5 dpf, two un-injected larvae and ten microinjected larvae that had developed normally were collected for a fragment length PCR analysis (Supp Fig. 12, Supp Table 3,4).

### Fragment length PCR analysis

Genomic DNA was extracted from each larva separately by incubating with 50 µL of proteinase K (Sigma-Aldrich, Munich, Germany) and lysis buffer (25 mM NaOH, 0.2 mM EDTA) (ratio 1:100) for 2 h at 55 °C and 10 min at 95 °C, followed by centrifugation at 3500 rpm for 3 min. For each CRISPR/Cas9-targeted site, the genomic region surrounding the targeted site was amplified by fluorescence PCR (Applied Biosystems 3730xl Genetic Analyzers, Thermo Fisher Scientific, Waltham, USA) and visualized using PeakScanner v1.0 (Thermo Fisher Scientific, Waltham, USA). The on-target efficiency of each single gRNA was subsequently quantified, and we selected one single gRNA per gene that showed a moderate or high on-target activity (Supp Figs. 11 and 12; Supp Tables 2, 3, 4 and 5). This required testing 32 single gRNAs across the 15 orthologues of 12 human genes, ranging from one single gRNA for most genes (*n* = 7) to five single gRNAs for *sec16b* (Supp Table 2).

### Generating CRISPR/Cas9 founders

Adult zebrafish with fluorescently labeled pancreatic beta cell nuclei (Tg:−1.2*insH2b*:mCherry) and hepatocytes (Tg:2.8*fabp10a*:GFP) were placed in breeding tanks overnight, with male and female fish separated by a transparent divider. In the morning, the divider was removed, and eggs were collected immediately after fertilization, followed by microinjection at the single-cell stage.

For the multiplexed screen in F_1_ larvae, genes were targeted in a multiplexed manner, by co-injecting single gRNAs and Cas9 mRNA against eight (Multiplex 1: *arid5b*, *lepr*, *mc4r*, *negr1*, *pcsk1*, *pomca*, *pomcb*, *sec16b*) and seven (Multiplex 2: *bdnf*, *irs1*, *irs2a*, *irs2b*, *sh2b1*, *sim1a*, *sim1b*) zebrafish orthologues of 12 human obesity genes. Aiming to limit the scope for genetic compensation^76^, zebrafish orthologues of the same human gene were always targeted together. At 5 dpf, larvae were optically screened for the presence of the fluorescently labeled pancreatic beta cell and liver transgenes using an EVOS FL Auto Imaging System microscope (Thermo Fisher Scientific, Waltham, USA). Transgene positive founder larvae were raised to adulthood.

### Generating F1 larvae

Adult CRISPR/Cas9 founder fish (multiplexed F_0_) were in-crossed; and at 5 dpf, the offspring (F_1_) were split based on the presence of both fluorescently labeled transgenes. Double positive larvae were transferred from an incubator to 1 L experimental tanks filled with 300 mL of water, at a density of 30 transgene positive or transgene negative larvae per tank. In each tank, larvae were overfed twice a day with 15 mg of dry food/feeding/30 larvae (Golden Pearls 50–100 μm particles, Alcester, UK), from 5 to 9 dpf. On day 7, tanks were cleaned by removing debris from the bottom and replenishing the volume back to 300 mL. Larvae without fluorescently labeled beta cells and/or liver were sacrificed and frozen for biochemistry-based analyses (see below) at 9 AM on day 10; while larvae carrying both fluorescently labeled transgenes were prepared for in vivo imaging followed by biochemistry-based analyses (see below).

### Singleplex approach

#### Designing and synthesizing CRISPR/Cas9 guide RNAs

For the singleplexed screen in CRISPR/Cas9 founders, controls were targeted at exon 4 of *kita*, while affected larvae were additionally targeted with up to two gRNAs at *pcsk1* or *sec16b*. Guide RNAs were designed using the CRISPOR v4.99 ^77^design tool to target early exons or functional domains with high efficiency, that is, to have: (1) high MIT specificity score; (2) a high Cutting Frequency Determination score; (3) t a high Azimuth in vitro score; (4) high out of frame values; (5) a high score for Lindel; and (6) a low number of predicted off-targets. Guide RNAs were synthetized as described in detail elsewhere^31^. In brief, equal volumes of 100 µM Alt-R^®^ crRNA and 100 µM Alt-R^®^ tracrRNA were mixed in Duplex Buffer (IDT, Coralville, Iowa, United States) and annealed (95 °C for 5 min, gradual cooling at −0.1 °C/sec to 25 °C, and 25 °C for 5 min). Next, 0.8 µL of 62 µM Cas9 protein stock (Alt-R^®^ S.p. Cas9 nuclease, v.3, IDT, Coralville, Iowa, United States) was gently mixed with 1 µL of 50 µM of each gRNA included in the injection mix, up to a total volume of 14 µL, to generate RNP complexes. RNP complexes targeting orthologues of the same human genes were combined in the same injection mix. RNP complexes were incubated at 37 °C for 5 min and 1 µL of 0.5% Phenol red (ThermoFisher, Waltham, USA) was added to the solution prior to micro-injections. Embryos were micro-injected as described above (see “*Designing and efficiency testing of CRISPR/Cas9 single guide RNAs*”). At 5 dpf, larvae were visually inspected, and those without anatomical abnormalities and with a minimal amount of pigment in the tail were transferred into 1 L experimental tanks at a density of 30 larvae/ 300 mL of water at a ratio of 70% affected larvae: 30% controls. Larvae were raised to 10 dpf as described above (see “*Generating F1 larvae*”) for in vivo image acquisition.

## Food intake

### Designing and synthesizing CRISPR/Cas9 guide RNAs

To examine the effects of mutations in 16 established obesity genes on food intake, fertilized eggs of AB fish were micro-injected at the single-cell stage. To generate affected larvae for a gene of interest, fertilized eggs were micro-injected with a duplex gRNA targeting the mast/stem cell growth factor receptor kita-encoding gene (*kita*) (Supp Table 12) and up to six gRNAs targeting early exons or domains of all orthologues of the human gene of interest (Supp Table 13) using the CRISPOR v4.99 ^77^ design tool as described above (see *Singleplex approach*). Targeting each gene in different locations with highly mutagenic duplex gRNAs increases the proportion of injected larvae that carry biallelic knockouts^31^. Controls were only targeted at the *kita* gene, using a different duplex gRNA with similar efficiency as used in the affected larvae. To reach the desired sample size, up to four rounds of micro-injections were performed and the gRNA targeting *kita* (i.e., target 1 or target 2) micro-injected in the affected larvae and controls were alternated each round.

At 5 dpf, larvae free from pigmentation were transferred to 1 L tanks, at a density of 30 larvae/300 ml of water in a ratio of 70% affected larvae: 30% controls, and at a maximum of 60 larvae/tank. Larvae were overfed with 16.3 mg/feeding/30 larvae twice per day using Zebrafeed, from day 5 to day 7. Between the morning and afternoon feedings of day 7, larvae were transferred to a clean tank with the same volume of water. In the evening of day 7, fluorescently labeled food was prepared by adding 75 µl of 2.0 μm polysterene microspheres (FluoSpheres carboxylate-modified microspheres, Invitrogen, Carlsbad, CA, USA) to 54 mg of Zebrafeed and 25 µL of deionized water. The mixture was dried at room temperature overnight and in the dark, and crushed into a fine powder the next morning. Image acquisition for food intake was performed at 8 dpf.

### Experimental design

#### Semi-automated, in vivo image acquisition for obesity-related traits

In the morning of 10 dpf, batches of 15 larvae were first transferred from their 1 L tank to 15 mL of fresh water. Next, larvae were transferred to a solution of 25 µM MDH in PBS, and kept in darkness for 30 min to selectively stain neutral lipids. The larvae were subsequently anesthetized using 0.04 mg/mL tricaine (MS-222, Sigma, Sweden), and aspirated into a borosilicate glass capillary using a Vertebrate Automated Screening Technology (VAST) BioImager (Union Biometrica Inc., Geel, Belgium) built on the stage of a Leica DM6000B automated fluorescence microscope with a DFC365 FX CCD camera (MicroMedic A/B, Stockholm, Sweden).

Twelve tomographic images were first acquired for each larva – one every 30° of rotation – using the brightfield camera of the VAST BioImager. Larvae were then positioned and oriented laterally to acquire a z-stack of 45 optical sections (∆Zi, Zi-1 = 1.5 μm) of the pancreatic islet using the microscope’s TXR filter and a 40X objective (Leica HCX APO L NA 0.80 W), with an exposure of 40 msec. After repositioning, two z-stacks of 35 optical sections each were acquired of the liver in lateral orientation – one from each side - using a 10X objective (Leica HCX APO L NA 0.30 U-V-I), L5 filter, and an exposure of 2 msec. Consecutively acquired z-stacks using a 405-filter were used to visualize MDH-stained lipids in adipocytes and liver, if present. Finally, optical sections of the dorsal aorta and caudal vein were acquired using a 20X objective (Leica HCX APO L NA 0.50 W) and L5 filter to visualize vascular lipid deposits, if present. An overview of the images acquired for obesity-related traits and their segmentation is shown in Supp Fig. 3. Exposure times were individually optimized across larvae for the lipid staining dye.

After completing image acquisition, the microscope sent a signal to the VAST BioImager to dispense the larva into a 96-well plate, followed by aspiration of the next larva for imaging.

### Quantifying LDLc, triglyceride, total cholesterol, glucose and protein content

Larvae negative for the fluorescently labeled transgenes marking pancreatic beta cell nuclei and/or liver were sacrificed by prolonged exposure to tricaine and ice at 9 AM on day 10. Larvae carrying both fluorescently labeled transgenes were first imaged (see above), before being sacrificed. Each larva was subsequently homogenized for 2 min at 1000 rpm (1600 MiniG-Automated homogenizer, Grammadate instrument, Uppsala, Sweden) in 88 µl of 1X PBS with two 1.4 mm zirconium beads (OPS Diagnostics). Following centrifugation (5 min, at 4 °C at 3500 rpm), 70 µL of the supernatant was collected for enzymatic assays, while the remaining pellet was used to isolate DNA for sequencing (see below). Within supernatant from each homogenized larva, LDLc (1E31, Abbott Laboratories, Abbott Park, IL, USA), triglycerides (7D74, Abbott Diagnostics, USA), total cholesterol (7D62, Abbott Laboratories, Abbott Park, IL, USA), and glucose (3L82, Abbott Laboratories, Abbott Park, IL, USA) concentrations were quantified on a BS-380 chemistry analyzer (Mindray, Shenzhen, China) and extrapolated to whole-body contents.

The final 12.5 µL of supernatant was transferred to a 96-well plate containing 12.5 µL of 1X PBS for protein quantification using the Pierce bicinchoninic acid (BCA) Protein assay kit (Thermo Fisher Scientific, Waltham, MA), at 562 nm using a Varioskan LUX Microplate Reader (Thermo Fisher Scientific, Waltham, MA, USA).

### Mutagenesis detection

DNA was extracted from the pellet of each larva using 50 µL of proteinase K (Sigma-Aldrich, Munich, Germany) and lysis buffer (25 mM NaOH, 0.2 mM EDTA) (ratio 1:100) for 2 h at 55 °C; 10 min at 95 °C; and centrifuging at 3500 rpm for 3 min at 4 °C.

### Library preparation for paired-end sequencing of F1 larvae

For each orthologue, the region surrounding the CRISPR/Cas9-targeted site was sequenced using paired-end Illumina sequencing on a MiSeq (2 × 250 bp). Forward and reverse primers were designed to include the targets in the center of each amplicon, which were PCR amplified and purified using magnetic beads (Mag-Bind PCR Clean-up kit, Omega Biotech Inc., Norcross, GA). A second PCR followed, in which Illumina Nextera DNA barcodes were added to 24 forward and 16 reverse primers, to uniquely label the PCR product of each well of a 384-well plate. Samples were purified with magnetic beads prior to sequencing at the SNP&SEQ Technology Platform in Uppsala.

During post-sequencing data management and quality control, *fastq *files were first demultiplexed into two files (forward and reverse) per well (i.e., larva) and amplified region (i.e., CRISPR/Cas9-targeted site). Next, a custom-written Pearl script was used to trim primer sequences from the reads. Forward and reverse reads were subsequently combined into a single read using PEAR^78^. FastX was then used for quality control^79^; STAR to align reads to the zebrafish reference genome (GRCz11)^80^; and SAMtools to convert .*sam* files into .*bam *files^81^. The .*bam* files served as input for a custom-written variant calling algorithm DIVaH (*Danio rerio *Identification of Variants by Haplotype), which identifies variants in the one or two most prominently observed reads of each CRISPR/Cas9-targeted site and larva. A larva was considered homozygous for the most prominently observed amplicon if that amplicon was observed at least 1.75-fold more frequently as compared with the second most prominently present allele. Otherwise, the larva was considered heterozygous. We next filtered variants outside a ± 30 bp window of the CRISPR/Cas9 cut site, and predicted the effect on protein function for variants inside the window using Ensembl’s Variant Effector Predictor (VEP)^33^ (Supp Fig. 11). In each larva, targeted site and allele, the variant with the highest likelihood of affecting protein function was retained, and weighed as 0.33, 0.66 or 1 for variants with a low (synonymous), moderate (missense) and high (frameshift and/or premature stop codon) predicted effect on protein function. Summing the weighted scores across the two alleles yielded a dosage for each targeted site in each larva.

### Fragment length analysis of single gene in F0 screening

We used fragment length analysis of the region flanking the CRISPR/Cas9 gene specific cut sites to distinguish between affected larvae and controls in the F_0_ screen for obesity-related traits. In the food intake experiment, we used a fragment length analysis of the region flanking the CRISPR/Cas9 cut sites of the two gRNAs targeting *kita*. Briefly, the regions of interest were amplified by PCR using M13-tailed forward primers in conjunction with pigtailed reverse primers^82^ (Supp Table 12, 13, 18, IDT, Coralville, Iowa, United States). The reaction mix was assembled using the OneTaq^®^ DNA Polymerase (M0480) (New England Biolabs, Ipswich, MA, USA): 2 µL of lysed Genomic DNA was combined with 2 µL of 5X OneTaq Standard Reaction Buffer, 0.2 µL of 10 mM dNTPs, 0.2 µL of 10 µM of M13, 0.2 µL of primer mix (5 µM of forward primer and 10 µM of reverse primer), 0.05 µL of OneTaq DNA Polymerase, and water to a total volume of 10 µL. The thermocycler profile followed the manufacturer’s instructions with minor adjustments: 94 °C for 30 s; 34 cycles: 94 °C for 30 s, 53–63 °C for 45 s depending on the primer pair, 68 °C for 30 s; final extension at 69 °C for 5 min.

The amplification of the segments surrounding the CRISPR/Cas9 cut sites in *kita* for the food intake experiment was performed by implementing the following modifications to the above method. A primer mix was assembled for each primer pair (Supp Table 12). Target 1 primer mix: 18 µL not-fluorescent forward primer, 2 µL 5ATTO550N-fluorescent forward, 20 µL reverse primer, and water up to 200 µL. Target 1 primer mix: 15 µL non-fluorescent forward primer, 5 µL 56-FAM-fluorescent forward primer, 20 µL reverse primer, and water up to 200 µL. Reactions and capillary electrophoresis were performed as described above.

The amplified product was diluted 5-fold and 1.5 µL of the sample was added to each well of a 96-well plate that was pre-loaded with 10 µL of Hi-Di™ formamide (ThermoFisher, Waltham, USA) and GeneScan™ −400HD ROX™ Size standard (ThermoFisher, Waltham, USA) (9.85 µL HiDi formamide and 0.15 µL Size standard per well). Of the 96 wells, eight wells did not contain samples and were used to quantify the background signal, 16 wells were loaded with DNA from un-injected sibling controls and were used to determine the size of the wild-type fragment sizes. The DNA was heated to 95 °C and rapidly cooled in ice prior to running the capillary electrophoresis on an Applied Biosystems^®^ 3730XL DNA analyzer (Applied Biosystems, Waltham, USA).

Peak Scanner version 2.0 (ThermoFisher, Waltham, USA) provided the fragment sizes and peak heights for each well analyzed by the DNA analyzer. An in-house R v.4.1.0. script was used to identify the wild-type and the mutant fragment sizes used to call larvae affected or controls for mutations in the obesity candidate gene. In brief, only the peaks of the channel corresponding to the fluorophore of the forward primers were considered. The background noise was determined by evaluating the height of the eight wells without DNA samples, and the peaks with a height ≤ the 95th quantile background peak height were excluded. Peaks with size outside the ± 50 bp window from the theoretical wild-type fragment size were also excluded^83^. The peak sizes of all samples were rounded to the full base pair. The wild-type peak was defined as the median fragment size across the highest peaks of the 16 wells with DNA of un-injected controls having a fragment size ± 1 bp of the theoretical wild-type fragment size. Across the samples with DNA of micro-injected larvae, non-wild-type peaks were assigned to be in-frame or frameshift, depending on whether the difference in base pair number between the peak and the wild-type peak was a multiple of 3 or not. For each sample, the relative peak areas of wild-type peaks, frameshift peaks, and in-frame peaks were calculated. Larvae were considered mutagenized if the relative wild-type peak area was < 0.50 and controls if the relative wild-type peak area was > 0.80. Larvae that fulfilled the criteria for at least one gene specific primer set (Supp Fig. 18) or for both *kita* target 1 and target 2 primer sets (Supp Fig. 19) were included in the statistical analysis.

### Image quantification

#### Body size

Using the twelve whole-body bright field images acquired by the VAST BioImager’s bright field camera, body length and area were quantified using a deep learning approach based on PyTorch. Briefly, a custom-written algorithm, trained on a dataset of thousands of images acquired in-house, distinguishes the larva from the capillary and background; draws a contour line around the silhouette of the larva; and estimates the body area as the number of pixels included within the contour line. Next, it draws a rectangle passing through the extreme points of the contour line (i.e., leftmost, rightmost, top, bottom) and estimates the length of the larvae. For larvae that were not completely within the field of view, the degree of missingness was quantified using the number of pixels in y at the site where the larvae intersected with the edge of the frame. Images with larvae overlapping the edge of the frame by ≥ 10 pixels were set to missing.

A maximal intensity projection method was applied to all optical slices within the z-stacks to generate a projected image, and fluorescence signals from the mCherry (pancreatic beta cell nuclei), GFP (liver), and MDH (lipids) were quantified using a custom-written pipeline.

### Vascular lipid deposition

Images of the vasculature in the tail region of the larva were analyzed using a deep learning approach based on PyTorch that segments stationary lipid deposits in the vasculature from the circulating lipids. Briefly, z-stacks were combined into a single maximum intensity projection and the algorithm was trained to recognize the circulation and hence, the ventral-caudal region of the tail, ranging from the lumen of the dorsal aorta to the ventral side of the caudal vein. The brightest spots were then segmented as lipid objects, and their area in the caudal vein and the dorsal aorta was quantified. Earlier studies showed that these reflect lipid deposits inside the vessel wall^69^.

### Liver size and hepatic lipid accumulation

Z-stacks of the liver were acquired in the lateral orientation from the right-hand side and the left-hand side. The images acquired from the left hand-side – closest to the largest lobe of the liver – were analyzed using a deep learning approach based on PyTorch. Briefly, the method combines the z-stacks in a maximum intensity projection; detects the GFP signal; draws a contour line; and estimates the area in pixels. Next, it detects and quantifies the brightest points in the detected area, representing the hepatic lipid droplets.

The images acquired from the right-hand side of the larva – closest to the smaller liver lobe – were manually annotated for the presence of lipid accumulation in adipocytes ventral-caudally of the swim bladder (i.e., abdominal or pancreatic) as well as in other anatomical regions (i.e., cranial, cardiac, appendicular). The images with lipids in adipocytes were also manually segmented to estimate the number of and area occupied by lipid droplets.

### Pancreatic beta cells

Images of the pancreatic islet were automatically analyzed using an algorithm based on deep learning. Briefly, the algorithm, trained on thousands of images, segments the nuclear mCherry signal. The algorithm estimates the volume, area, and diameter of the islet of Langerhans. It also counts the number of beta cell nuclei. For each segmented beta cell nucleus, it estimates the volume and the average and total fluorescence intensity across the z-stack, i.e., in 3D. The nuclear volume at least in part reflects the cell cycle stage, while the beta cell average and total nuclear fluorescence intensity are used as proxy measures for beta cell insulin expression.

### Food intake

On the morning of day 8, batches of 8 larvae/tank were transferred to a beaker with 80 mL of fresh water for 1 h. Larvae were subsequently exposed to fluorescently labeled food for 1 h^84^. Full body images and z-stacks (50 images at 1.5 μm focal distance) of the gastrointestinal tract were recorded using a 4X objective (Leica, HI PLAN NA 0.10 POL), until 1 h after the end of feeding (Supp Fig. 16). To avoid bias by batch and time of imaging, larvae from each tank were imaged alternatingly and the time at imaging was recorded. Larvae were identified as being affected or controls after the experiment (see “*Fragment length PCR analysis*”).

### Statistical analysis

With the exception of lipid deposition in adipocytes and vasculature, all outcomes were normally distributed. For each normally distributed outcome, we first set observations outside the mean ± 5 × SD interval to missing (e.g., 4 and 7 observations in total for Multiplexes 1 and 2). Normally distributed outcomes were then inverse-normally transformed to a mean of 0 and a standard deviation of 1, so betas and standard errors of linear regression models can be interpreted as z-scores and effect sizes can be compared across outcomes. For each outcome, the effect of exposures was then examined using multiple linear regression models, adjusting for batch (multiplexed F_1_ screen), tank (F_0_ screen) and time of day at imaging (Model 1); or additionally for body length and dorsal area normalized for body length (Model 2, for appropriate outcomes). Results from Model 2 should be interpreted with caution, since adjusting for body size may introduce collider bias or spurious results if effects on body size are secondary to direct effects on the outcome. For Models 1 and 2, effects were additionally adjusted for trait-specific covariables where appropriate (i.e., body length for dorsal and lateral body area). Given its distribution, effects of exposures on vascular lipid deposition were examined using a negative binomial regression analysis, while genetic effects on the presence or absence of lipids in adipocytes were examined using logistic regression. In the multiplexed genetic screen, effects were examined for mutations in all genes simultaneously, with larvae from both multiplexes pooled, the number of mutated alleles at untargeted sites set to zero, and with – for each gene – effects adjusted for the number of mutated alleles at simultaneously targeted genes, weighted by the predicted effect on protein function based on Ensembl’s VEP.

In the multiplexed genetic screen (F_1_ generation), the effect of mutations was examined using one of two models: (1) A homozygous-like model, preferably by comparing outcomes between larvae with frameshift- and/or premature stop codon-introducing mutations (FS/PS mutations) in both alleles and larvae free from CRISPR/Cas9-induced mutations in the gene (dichotomous variable, 2 vs. 0 mutated alleles), for genes with at least 10 larvae in both genotype groups (for *mc4r*, *negr1*, *pcsk1*, *sec16b*, *irs2b*, *sh2b1*, *sim1b*); or, alternatively, (2) using an additive model. In this case, the exposure is a “dosage” variable containing information -for each gene- on whether the larva carried 0, 1 or 2 mutated alleles, weighted by their predicted effect on protein function using VEP (0 = no variant; 0.33 = synonymous, 0.66 = missense 1 = frameshift or premature stop codon). Each final “dosage” variable was used as a continous independent variable in the regression model and could have values from 0 to 2 with 0.33 increments. This model was used for *arid5b*, *lepr*, *pomca*, *pomcb*, *bdnf*, *irs1*, *irs2a*, *sim1a*. In addition, effects of mutations were examined across all targeted sites simultaneously – within each multiplexed line – as a within-gene sum of the number of mutated alleles, weighted by the predicted effect of each mutation on protein function, for each of the eight or seven targeted sites.

For most of the study, we generated dedicated datasets to examine genetic effects on one adiposity traits per dataset (i.e., food intake or lipid accumulation in adipocytes in the F0 experiments). This does not warrant adjusting for multiple testing. Further, a principal component analysis shows that in multiplex 1 and 2 data, 54% of the overall phenotypic variance is explained by only two components. Hence, a Bonferroni corrected alpha of 0.025 would be appropriate when evaluating genetic effects on multiple cardiometabolic traits. Adjusting for multiple comparisons removes 5 of the 20 significant genetic effects on cardiometabolic outcomes. This does not affect the conclusion that we can use the model system to identify genetic effects on cardiometabolic traits.

The statistical analysis was performed using STATA MP v16, and graphs were generated using R and R studio v.2022.07.2. A detailed description of the different regression models used for the statistical analysis can be found in Supp Table 21. All methods were performed in accordance with the relevant guidelines and regulations. The study is reported in accordance with ARRIVE guidelines.

## Electronic supplementary material

Below is the link to the electronic supplementary material.

Supplementary Material 1

Supplementary Material 2

## Data Availability

The datasets generated and/or analysed during the current study are available on GitHub (https://github.com/denHoed-Lab/Obesity_Zebrafish_Data_Scripts). The custom-written variant calling algorithm (i.e., DIVaH - Danio rerio Identification of Variants by Haplotype) is also available on GitHub (https://github.com/denHoed-Lab/DIVaH). All methods were performed in accordance with the relevant guidelines and regulations. The study is reported in accordance with ARRIVE guidelines.
