# Supplementary material for: Functionally characterizing obesity-susceptibility genes using CRISPR/Cas9, in vivo imaging and deep learning
Source: Sci Rep. 2025 Feb 13;15:5408. doi: 10.1038/s41598-025-89823-2 (PMC11825957; doi:10.1038/s41598-025-89823-2)
Supplement: Supplementary file 1 — Supplementary Material 1 [file 41598_2025_89823_MOESM1_ESM.docx]

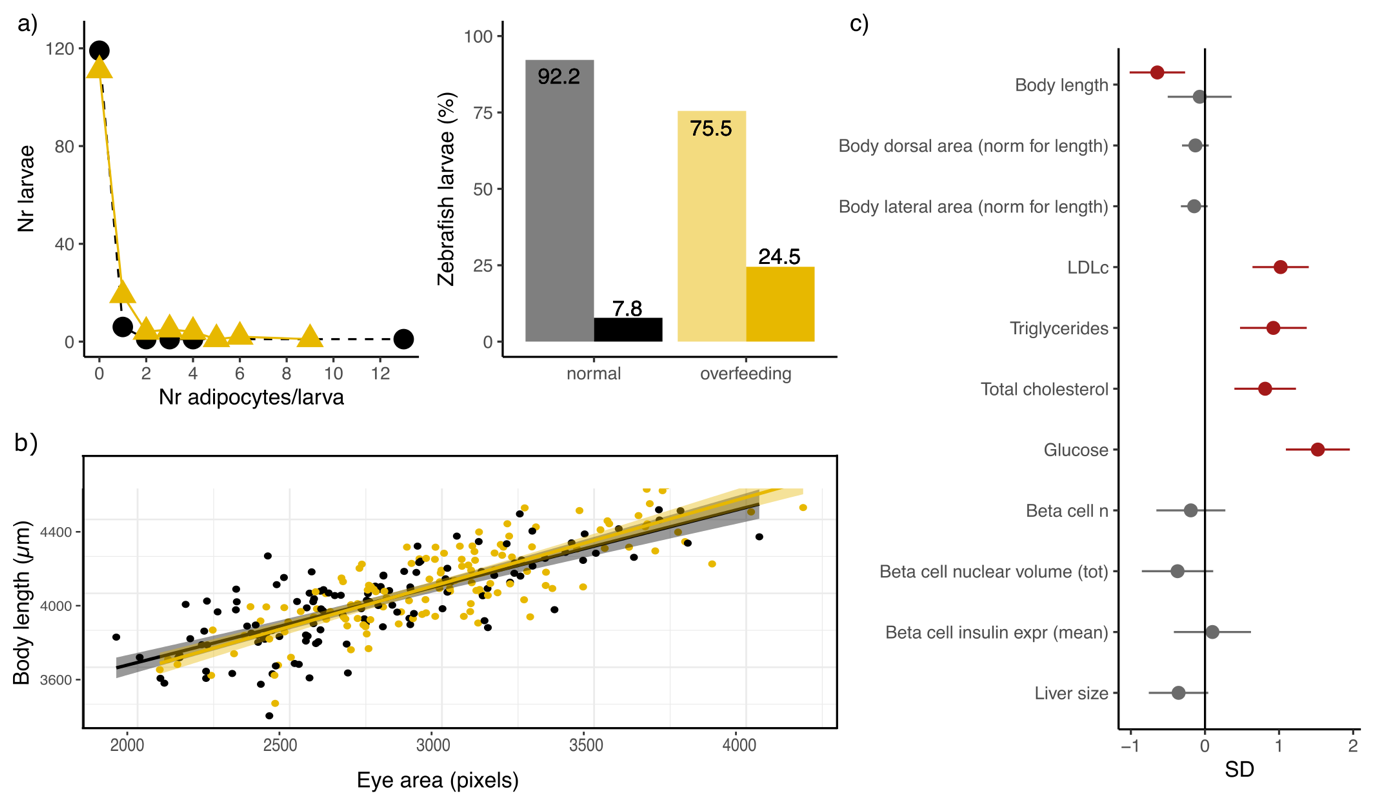


**Supplementary Figure 1. The effect of 5 days of overfeeding on lipid accumulation in adipocytes.** **a-b**) overfeeding in yellow; control amount in black; **a)** Number of larvae without (lighter color) or with at least one adipocyte (darker color); **c)** dots and error bars show effect sizes and 95% confidence intervals for overfed larvae vs. control larvae fed 3x less. Effects are adjusted for batch and time of day at imaging. Effect sizes with P<0.05 are shown in red.


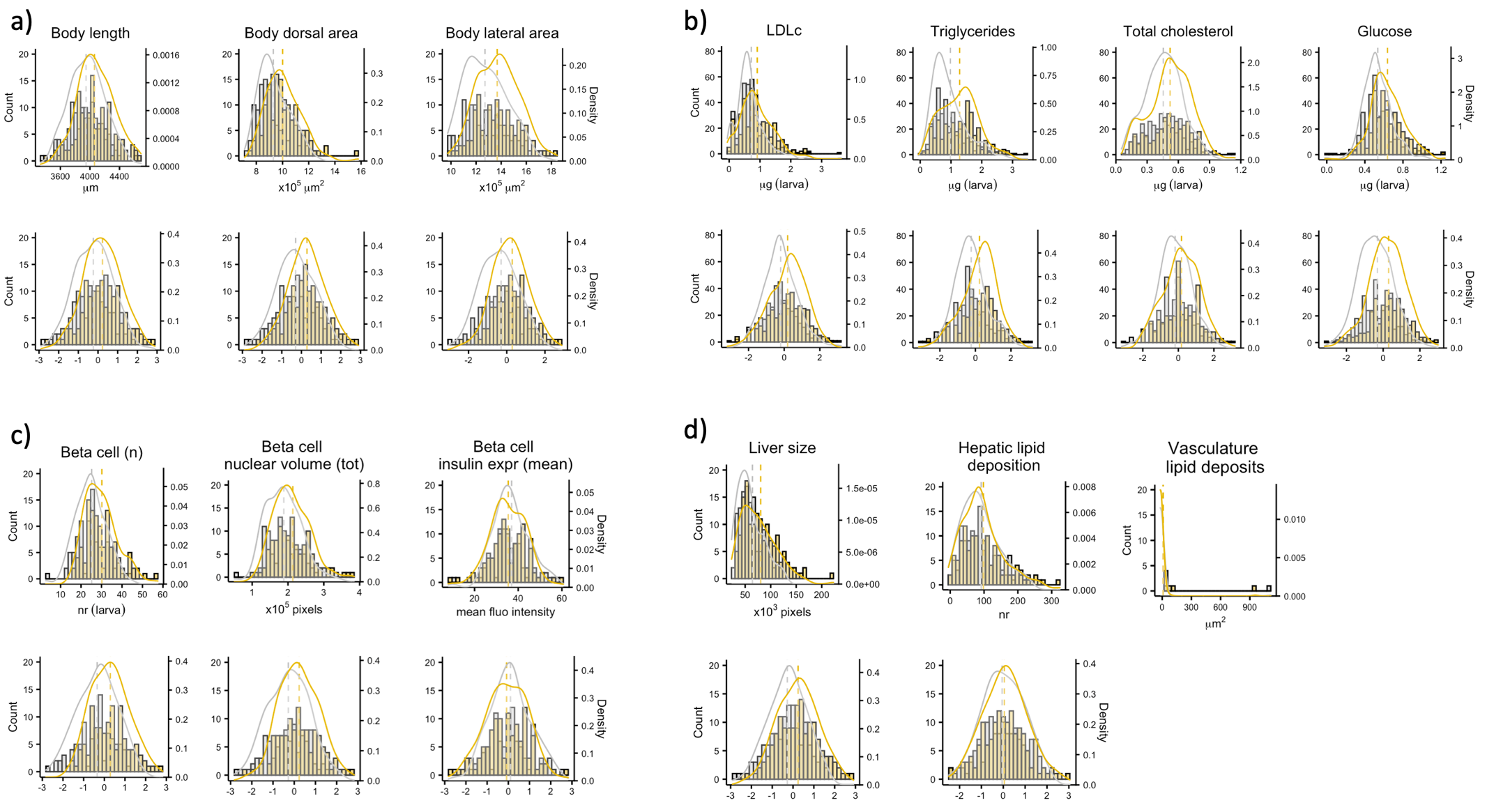


**Supplementary Figure 2. Effect of 5 days of overfeeding on image- and biochemistry-based cardiometabolic traits in 10-day-old zebrafish larvae.** Distributions of **a)** body size traits; **b)** cholesterol and glucose-related whole-body contents; **c)** beta cell traits; **d)** liver traits and vascular lipid deposition. Distributions are shown for overfed larvae (yellow) and for control larvae fed 3x less (grey) separately. Raw values (top) and residuals adjusted for batch and time of day at imaging (bottom) are shown.

**
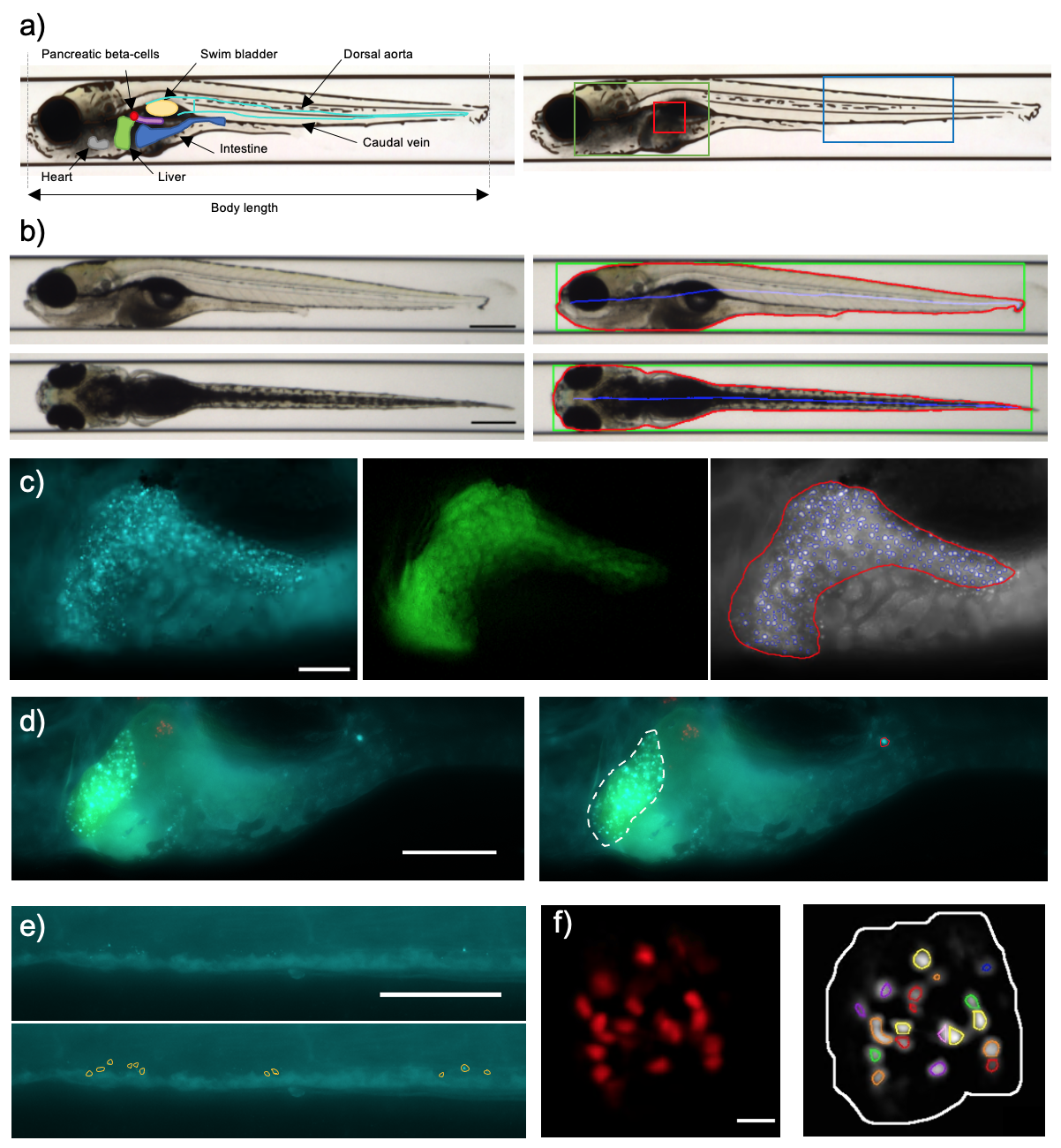
**

**Supplementary Figure 3. Visualizing and quantifying image-based cardiometabolic traits in live, 10-day-old zebrafish larvae using fluorescence microscopy and deep learning. a)** General anatomy of a zebrafish larva at day 10 (left) and regions of interest for the image acquisition (right) of lipid deposition in the liver and adipocytes (green box); vascular lipid deposition (blue box); number, nuclear volume, and average nuclear fluorescence intensity of beta cells (red box) (scale bar = 200 μm); **b)** whole-body images of a zebrafish larva (left) in lateral (upper) and dorsal orientation (lower) and their segmentation (right) for estimation of body length, lateral area, and dorsal area (scale bar = 100 μm); **c)** hepatic lipid deposition is stained with a lipophilic dye (monodansylpentane) that emits a fluorescence signal in the blue end of the spectrum in larvae with transgenically expressed, fluorescently labeled hepatocytes (Tg:2.8*fabp10a*:GFP) and beta cell nuclei (Tg:-1.2*insH2B*:mCherry). The GFP-labelled transgene allows visualization and quantification of liver size and lipid deposition in the liver (scale bar = 10 μm); **d)** lipids stored in adipose tissue are segmented (right) to quantify their number and area (scale bar = 200 μm); **e)** maximal projection of the circulating lipids and vascular lipid deposits in the intersegmental vessels feeding into the caudal vein which are automatically segmented to quantify their combined area (scale bar = 100 μm); **f)** visualization and segmentation of the nuclei of pancreatic beta cells, from which the beta cell number, nuclear volume and nuclear insulin expression can be quantified within the region of interest (white line) (scale bar = 10 μm).


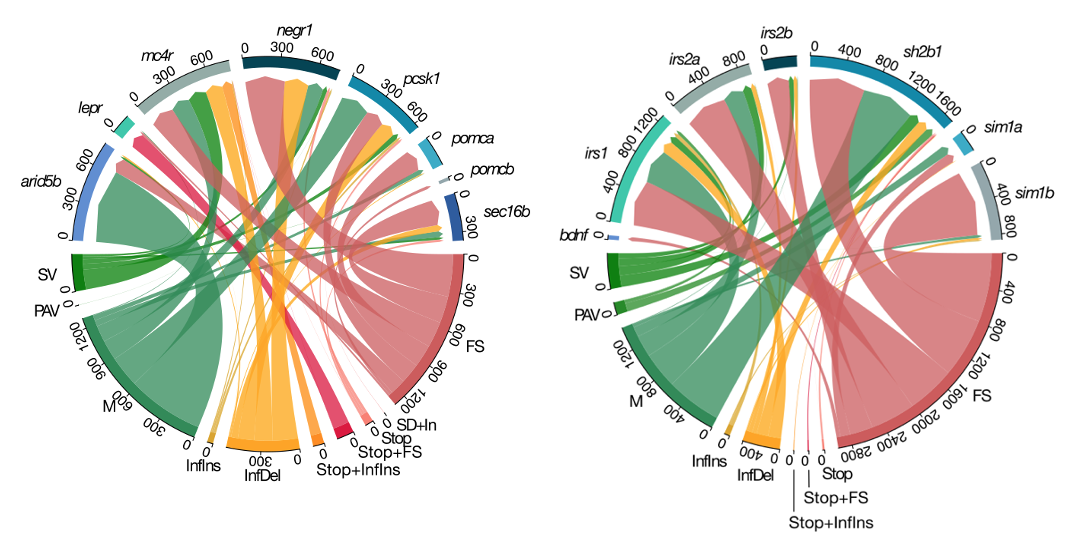


**Supplementary Figure 4. Distribution of mutation types induced by CRISPR/Cas9 across the eight genes targeted in Multiplex 1 (left) and the seven genes targeted in Multiplex 2 (right).** Color-coded variants with a high (red), moderate (yellow) and low (green) anticipated impact on protein function. The grid shows the number of alleles affected by each type of mutation. In both multiplexes, most variants detected at CRISPR/Cas9-targeted sites in the F_1_ generation were predicted to have a highly deleterious impact on protein function.

Mutation types: FS = frameshift variant; SD+In = splice donor variant, coding sequence variant, intron variant; Stop = stop gained; Stop+FS = stop gained, frameshift variant; Stop+InfIns = stop gained, inframe insertion; InfDel = inframe deletion; InfIns = inframe insertion; M = missense variant; PAV = protein altering variant; SV =synonymous variant.

**
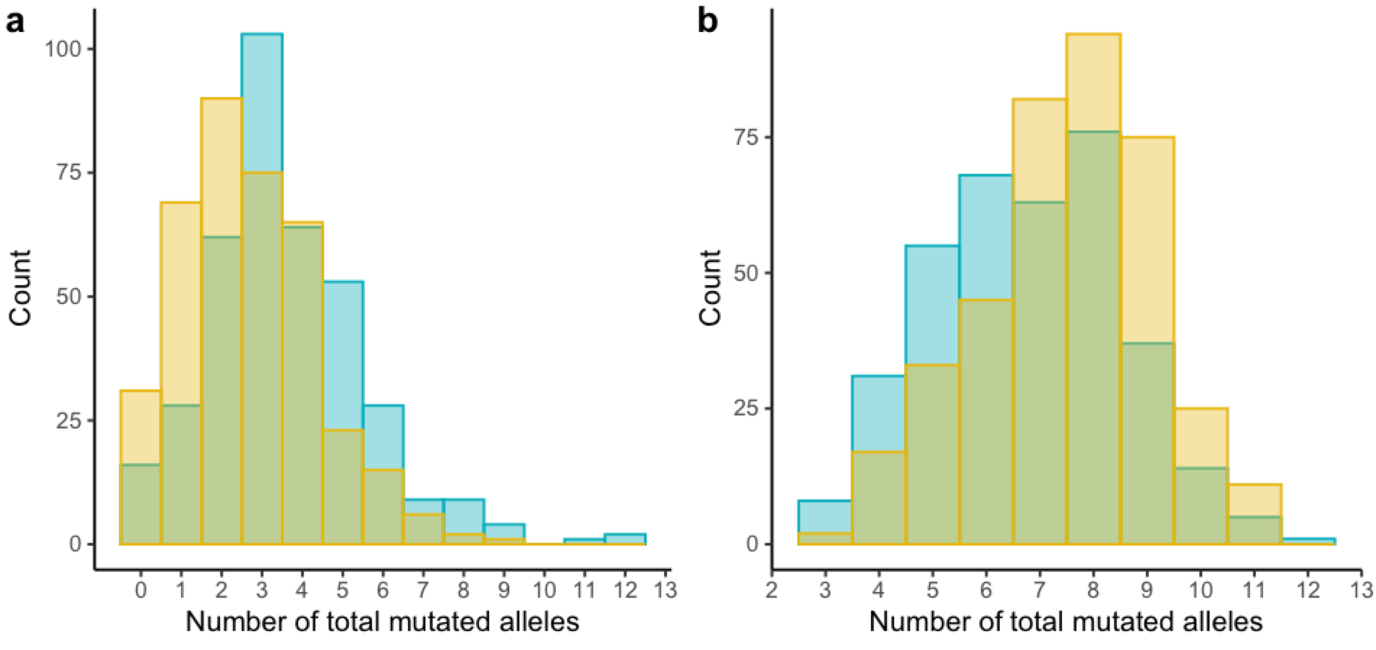
**

**Supplementary Figure 5. Distribution of the total number of mutated alleles across the eight genes targeted in Multiplex 1 (a) and across the seven genes targeted in Multiplex 2 (b).** Histograms are stratified for larvae that have been imaged (yellow) or used for biochemistry-based analyses (blue).

Multiplex 1: *arid5b*, *lepr*, *mc4r*, *negr1*, *pcsk1*, *pomca*, *pomcb*, *sec16b*;

Multiplex 2: *bdnf*, *irs1*, *irs2a*, *irs2b*, *sh2b1*, *sim1a*, *sim1b*.


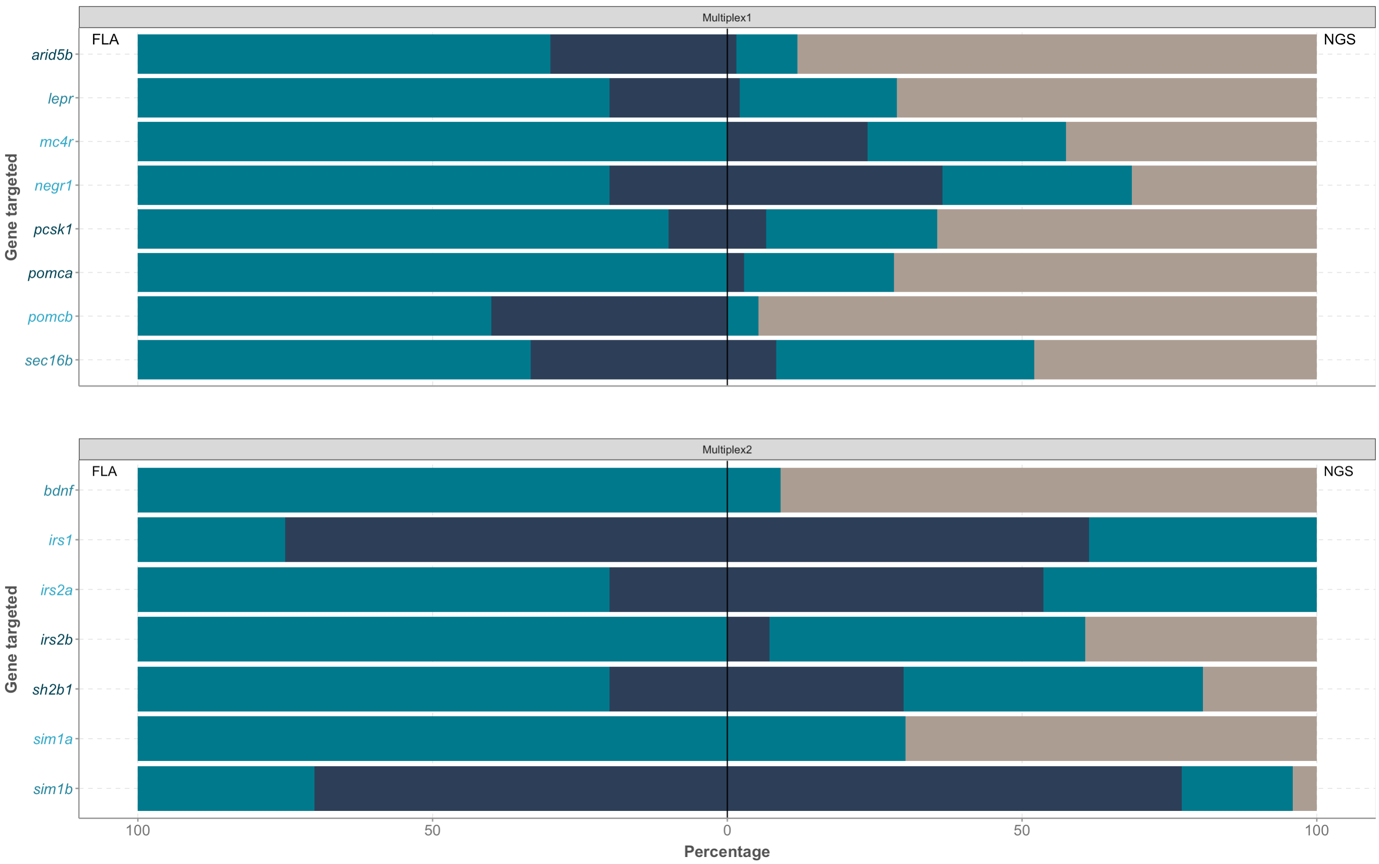


**Supplementary Figure 6. Comparison of the CRISPR/Cas9 single gRNA mutagenic efficiency in test injections and in the F_1_ screen.** Diverging stacked bar plot showing the performance of the single gRNA when tested for efficiency using fragment length analysis (FLA) in CRISPR/Cas9 founders (left of zero line) and the mutant allele frequency in F_1_ larvae using paired-end sequencing (2x250 bp, right of zero line). The colors of the bar indicate the percentage of larvae in which the CRISPR/Cas9-induced mutations had low (grey), moderate (light blue), or high efficiency (dark blue). The colors of the labels on the y-axis (left) represent the *in-silico* prediction of mutagenic effect on protein function using Ensembl’s Variant Effector Predictor (VEP) (*e.g.*, light blue = medium efficiency, dark blue = high efficiency).

**Supplementary Figure 7. Association of CRISPR/Cas9 activity in test injections vs. mutant allele frequency in the F_1_ larvae.** The percentage of non-wild type (Wt) peaks in test injections quantified using a fragment length analysis (FLA) as a function of the mutant allele frequency (MAF) in the F_1_ larvae that were phenotypically characterized and paired-end sequenced (2x250 bp, NGS) at 10 days post fertilization. The color indicates if the allele deviates from Hardy-Weinberg equilibrium (HWE) (pink) or not (blue) and if the number of larvae with two mutated alleles in the F_1_ larvae is lower than expected (dark pink), which would suggest that homozygous mutants are less viable. The symbol shape indicates if genes were targeted in Multiplex 1 (dot) or Multiplex 2 (triangle).


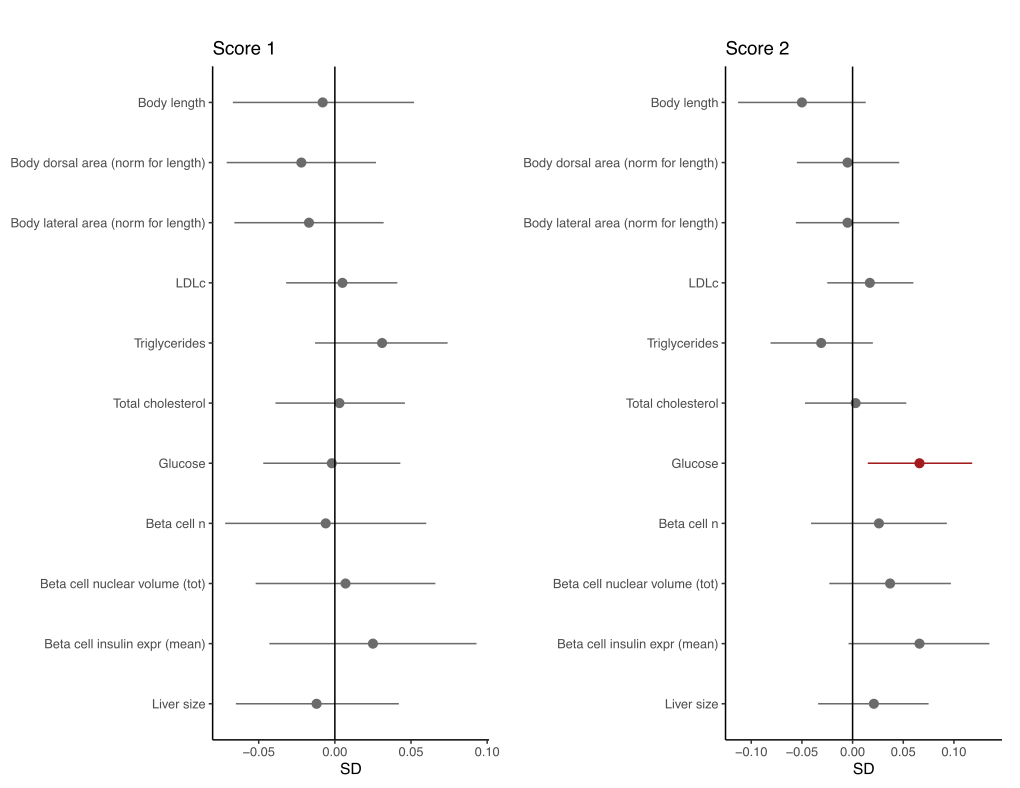


**Supplementary Figure 8. Associations of image- and biochemistry-based cardiometabolic traits with genetic burden scores across eight (Multiplex 1, left) and seven (Multiplex 2, right) targeted obesity genes in 10-day-old F_1_ larvae, i.e., offspring of CRISPR/Cas9 founders.** Dots and error bars show effect sizes and 95% confidence intervals for each additional CRISPR/Cas9-mutated allele, weighted by their predicted effect on protein function based on Ensembl’s variant effect predictor (high=1; moderate=0.66; low=0.33). In each Multiplex, image-based results are based on data from up to 384 larvae; and biochemistry-based results (LDLc, triglyceride, total cholesterol, and glucose levels) are based on data from up to 384 other larvae. Effects are adjusted for multiplex, batch, time of day at imaging, and the weighted effect of mutations in genes targeted in the other multiplex.

Multiplex 1: *arid5b*, *lepr*, *mc4r*, *negr1*, *pcsk1*, *pomca*, *pomcb*, *sec16b*;

Multiplex 2: *bdnf*, *irs1*, *irs2a*, *irs2b*, *sh2b1*, *sim1a*, *sim1b*.


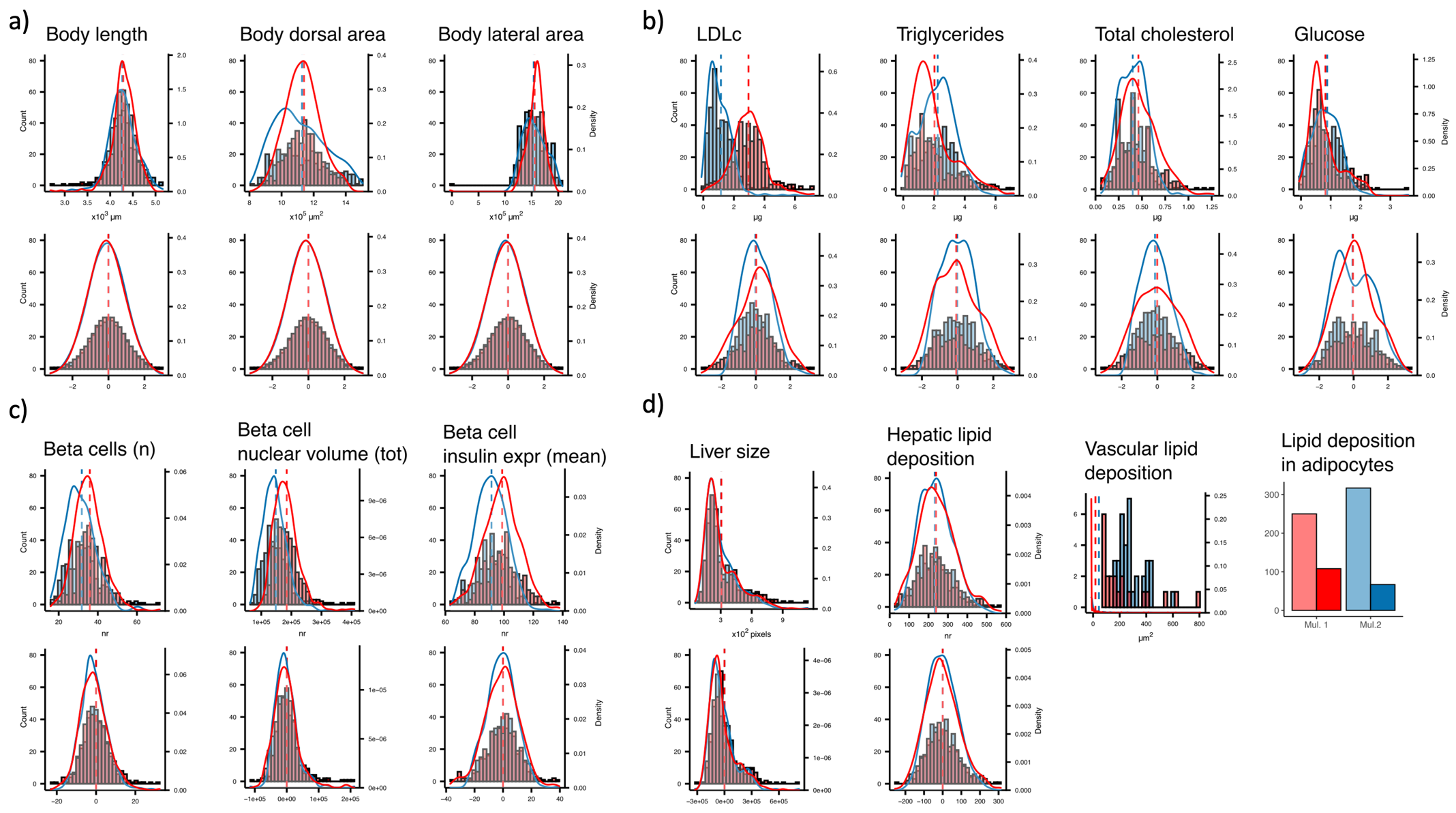


**Supplementary Figure 9. Distribution of raw values (upper) and residuals (lower) of the image- and biochemistry-based cardiometabolic traits quantified in Multiplexes 1 (red) and 2 (blue).** Distributions of **a)** body size traits; **b)** cholesterol and glucose-related whole-body contents; **c)** beta cell traits; **d)** liver traits, vascular lipid deposition, and number of larvae with (dark color) and without (light color) lipid accumulation in adipocytes.

1. *arid5b*


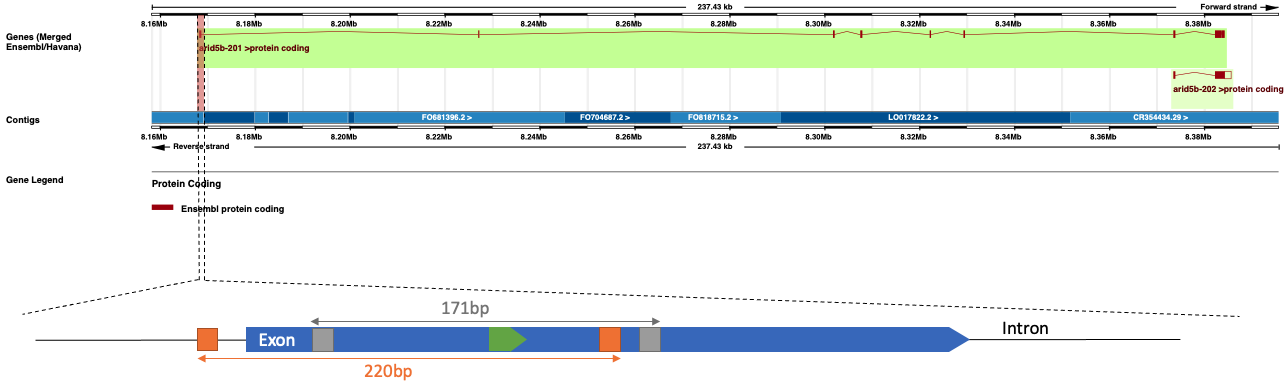


1. *lepr*


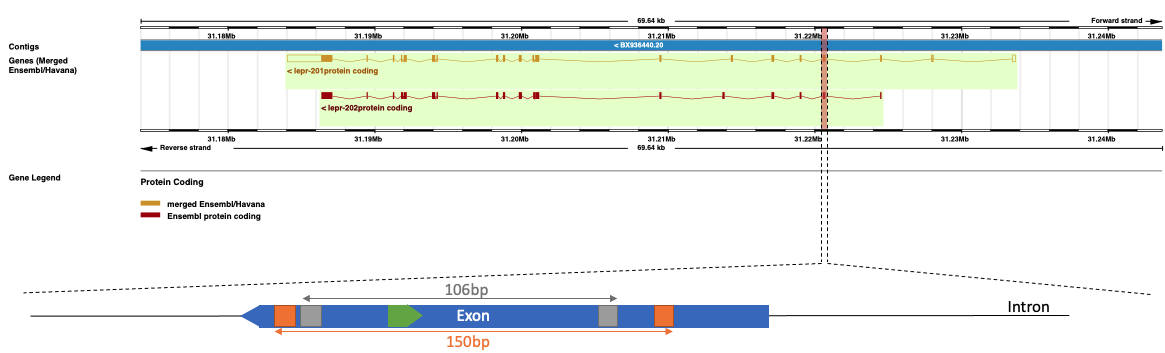


1. *mc4r*


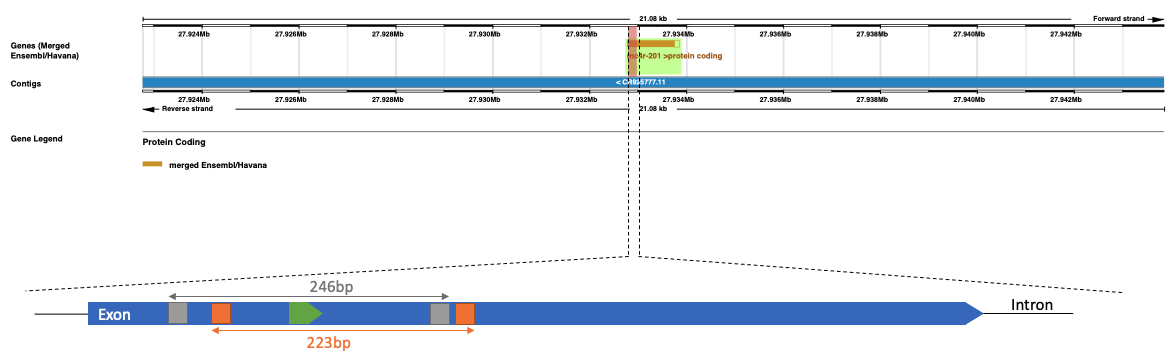


1. *negr1*


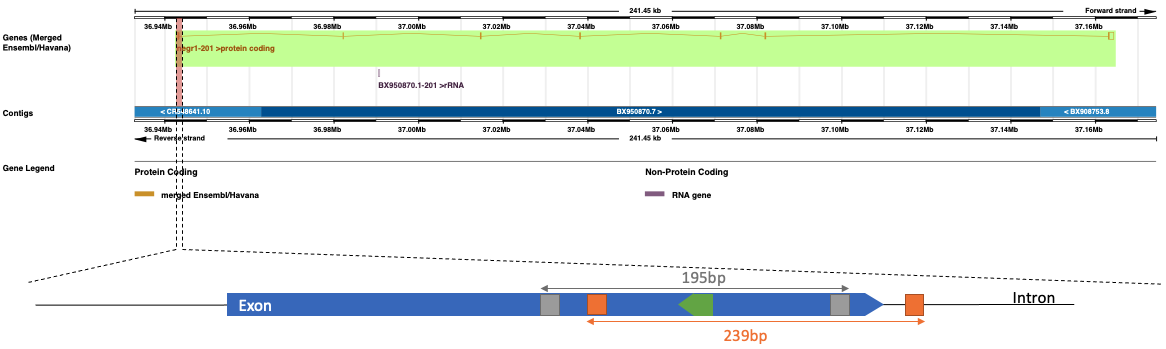


1. *pcsk1*


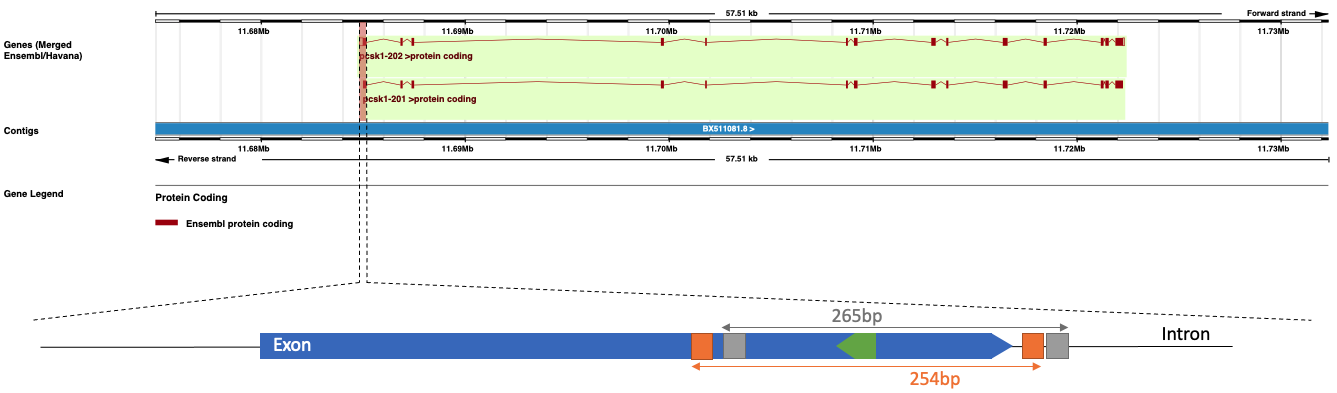


1. *pomca*


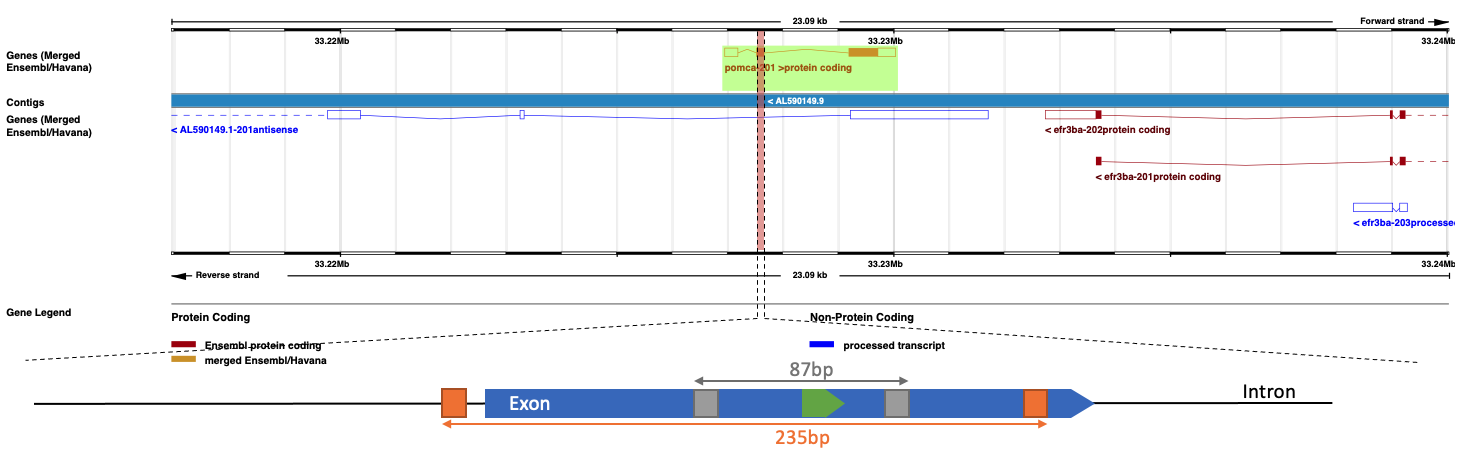


1. *pomcb*


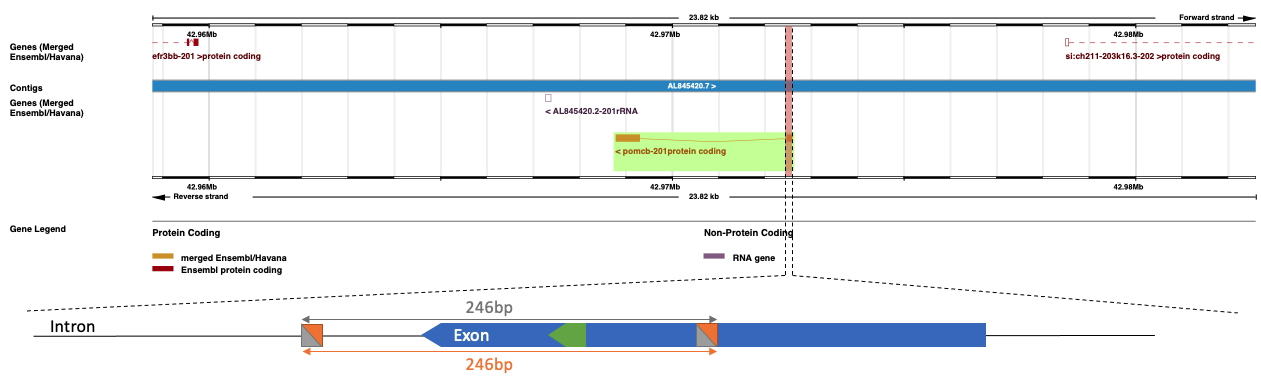


1. *sec16b*


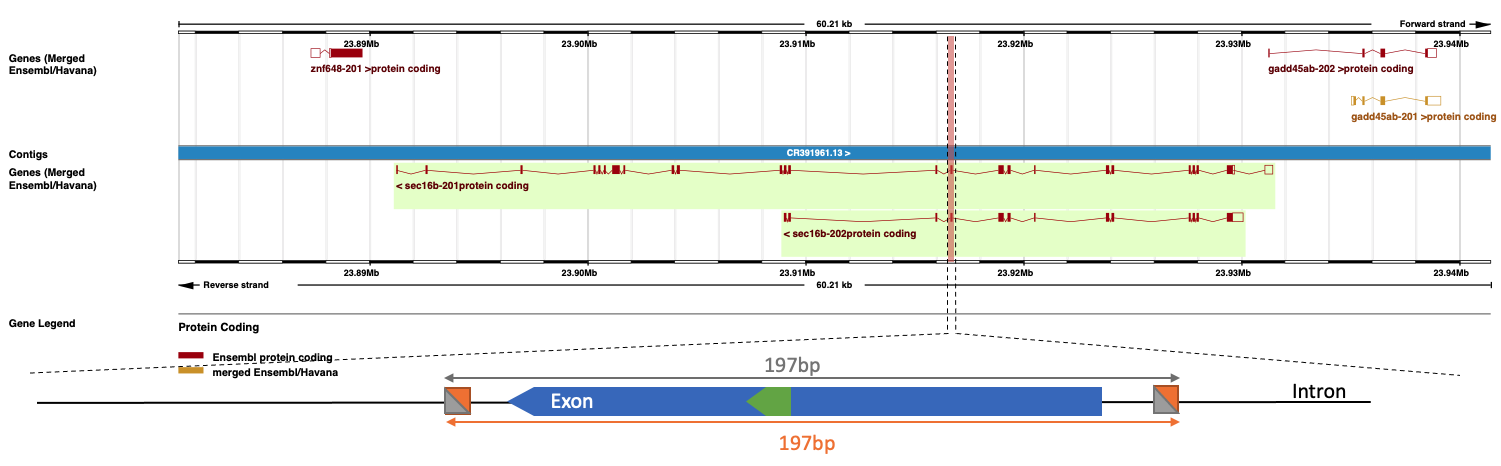


1. *bdnf*


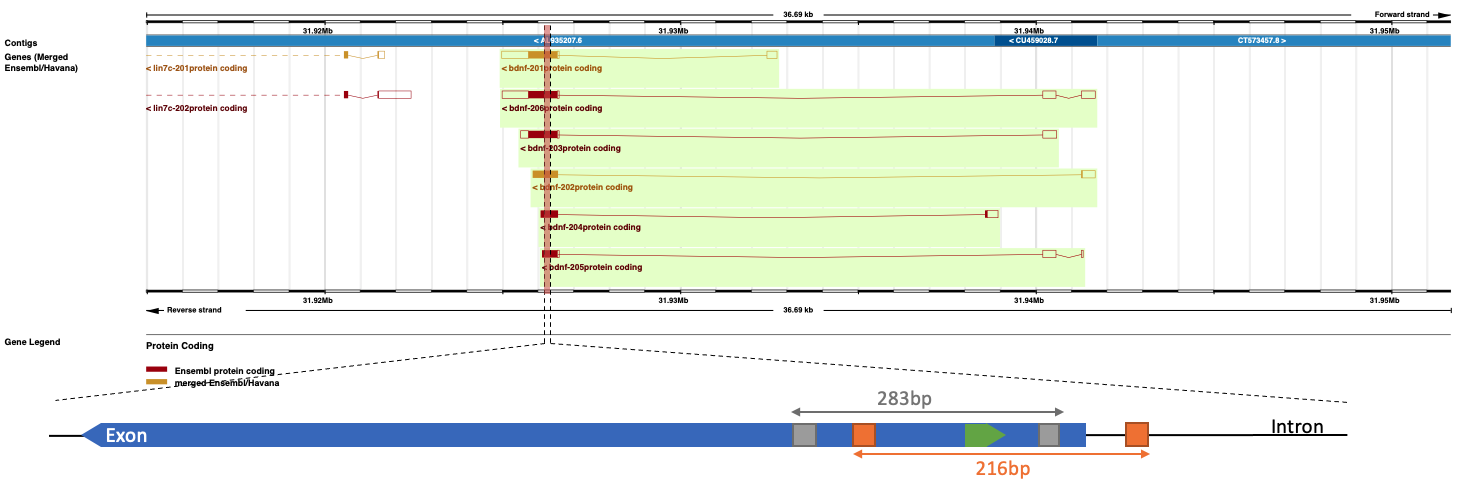


1. *irs1*


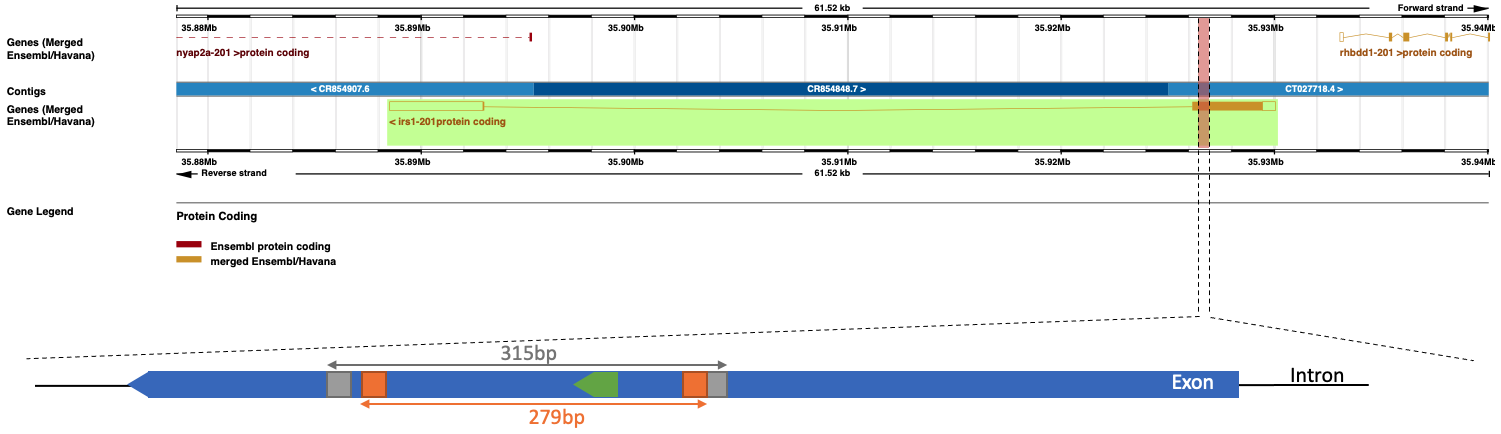


1. *irs2a*


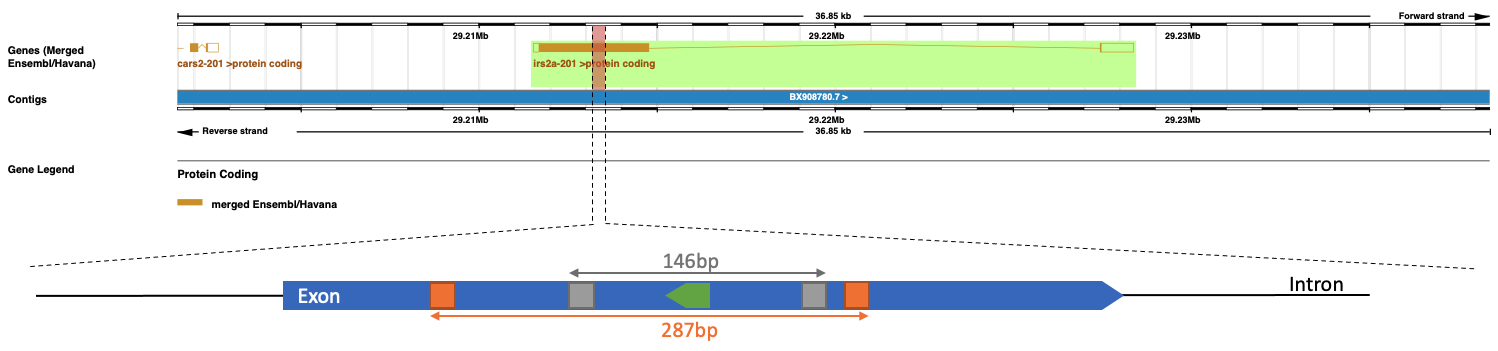


1. *irs2b*


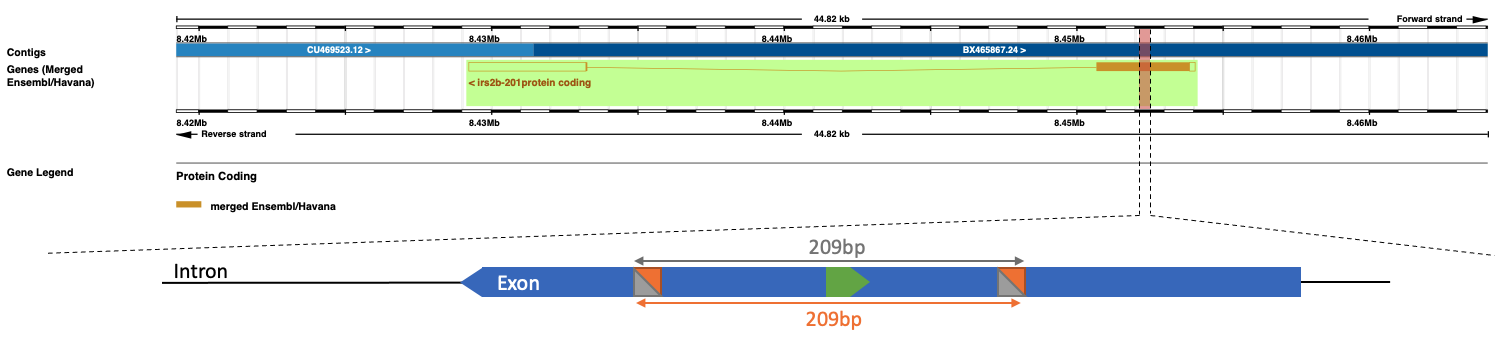


1. *sh2b1*


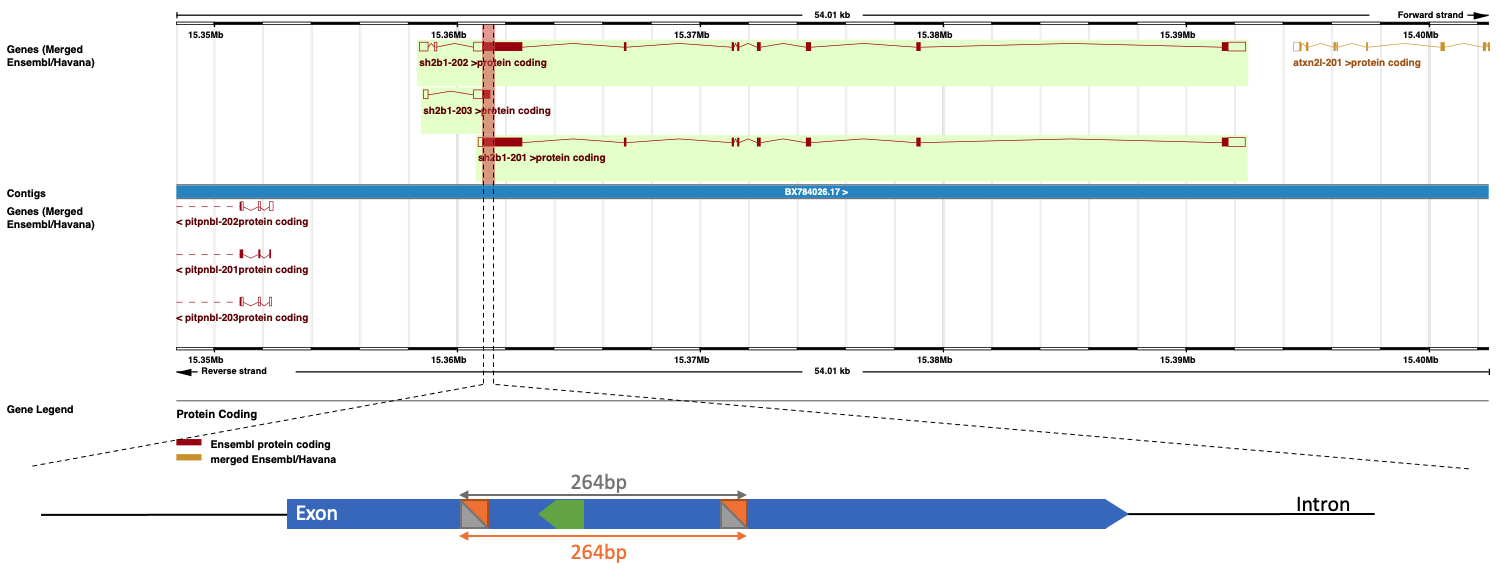


1. *sim1a*


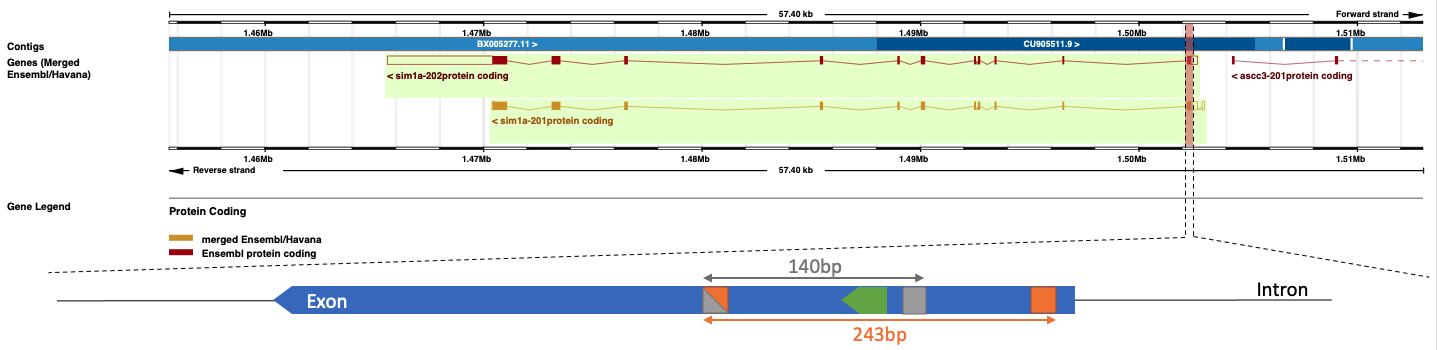


1. *sim1b*


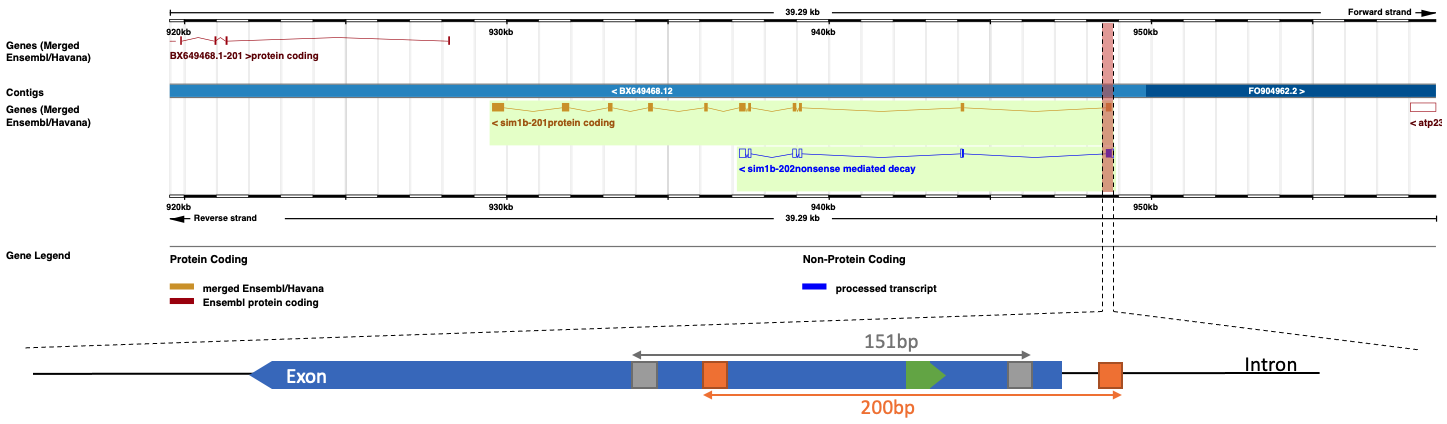


**Supplementary Figure 10.** **Transcripts of the 15 zebrafish genes targeted in multiplex and information on the CRISPR/Cas9 targeted sites.** Protein coding transcripts are shown in red (Ensembl protein coding) or gold (merged ensemble/Havana) (Images adapted from *Ensembl*) and the exon targeted using CRISPR/Cas9 is highlighted by a red box. The dotted lines represent a closer view of the CRISPR/Cas9-targeted region: the blue arrow indicates the direction of transcription; the green arrow shows the location and direction of the single gRNA. The boxes show the location of primers for the fragment length analysis (F_0_, orange) and for paired-end sequencing (F_1_, grey).

a) *arid5b* b) *lepr*

*c) mc4r* d) *negr1*

** **

e) *pcsk1* f) *pomca*

** **

g) *pomcb* h) *sec16b*

** **

i) *bdnf* j) *irs1*

 **

k) *irs2a* l) *irs2b*

**

m) *sh2b1* n) *sim1a*

** **

o) *sim1b*

**

**Supplementary Figure 11.** **Efficiency of the selected CRISPR/Cas9 single gRNAs targeting the 15 zebrafish genes in multiplex.** The bar plot shows the size (in percentage) of the relative area of peaks that are estimated to be wild-type, in-frame mutations, or frameshift mutations in the test-injected larvae (Test) and in two un-injected controls (Con). The donut plots show the overall percentage of wild-type (grey), in-frame (green), and frameshift (blue) peaks across all test-injected larvae (upper); the percentage of all test-injected larvae with only wild-type (grey), wild-type and extra (light pink), and only extra peaks (dark pink) (lower)

1. *arid5b*

*
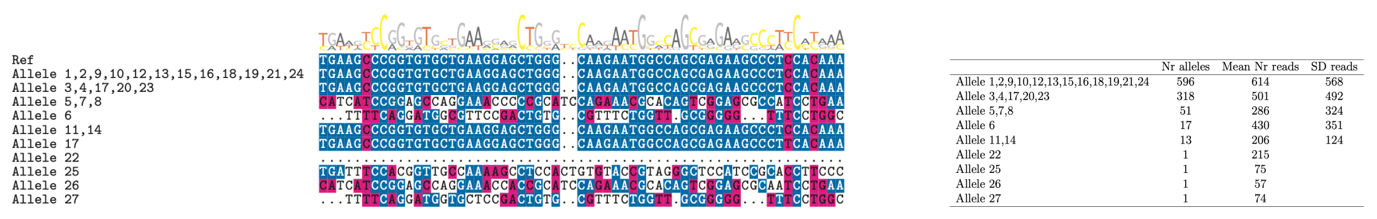
*

1. *lepr*


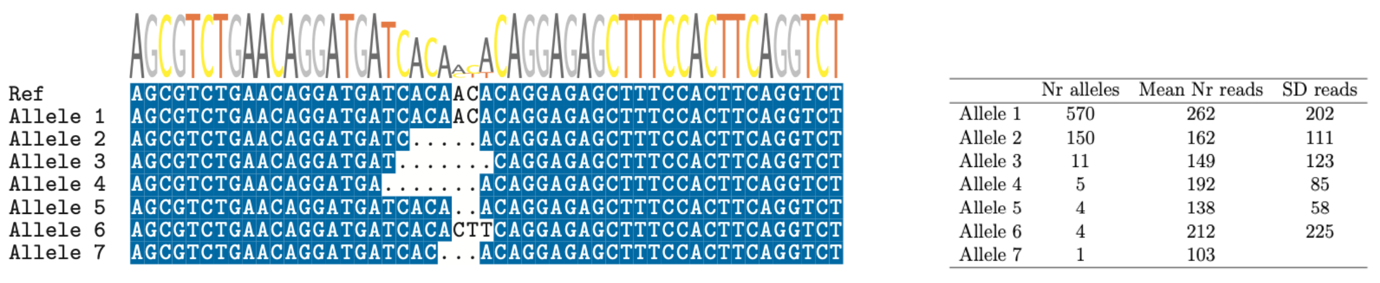


1. *mc4r*

*
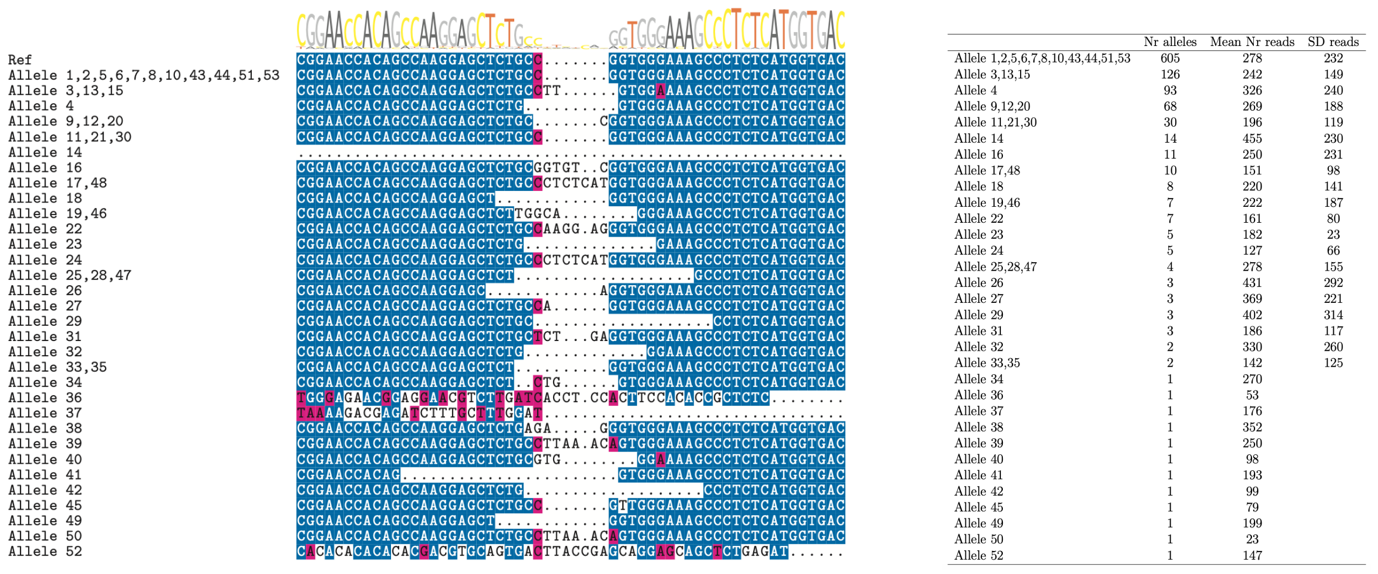
*

1. *negr1*

*
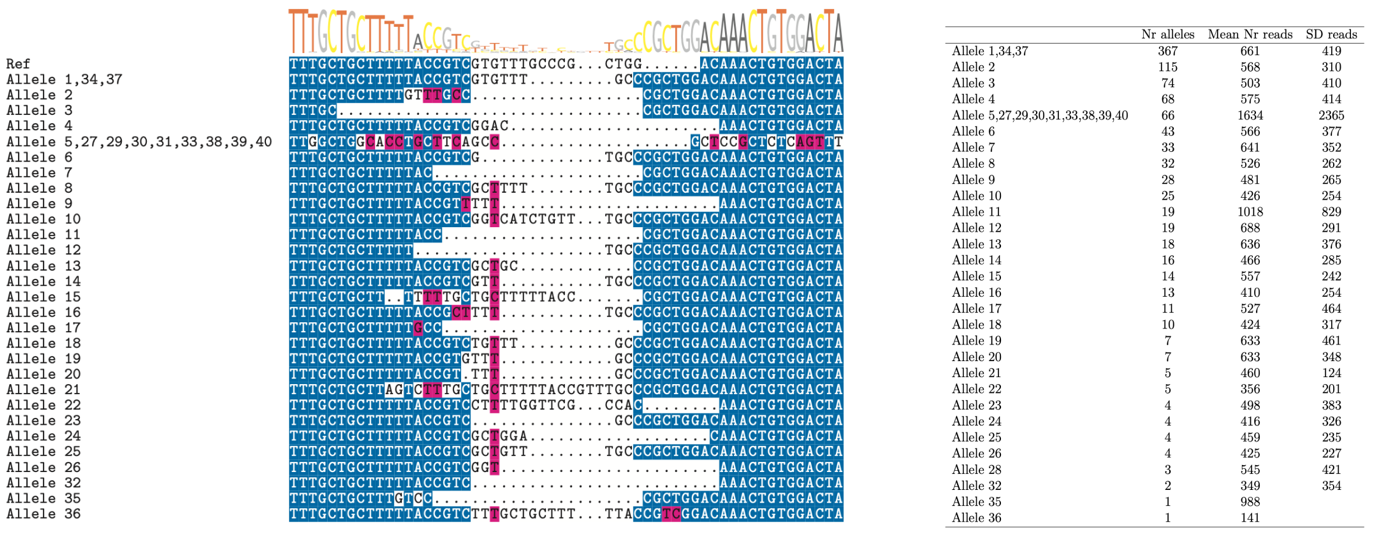
*

1. *pcsk1*

*
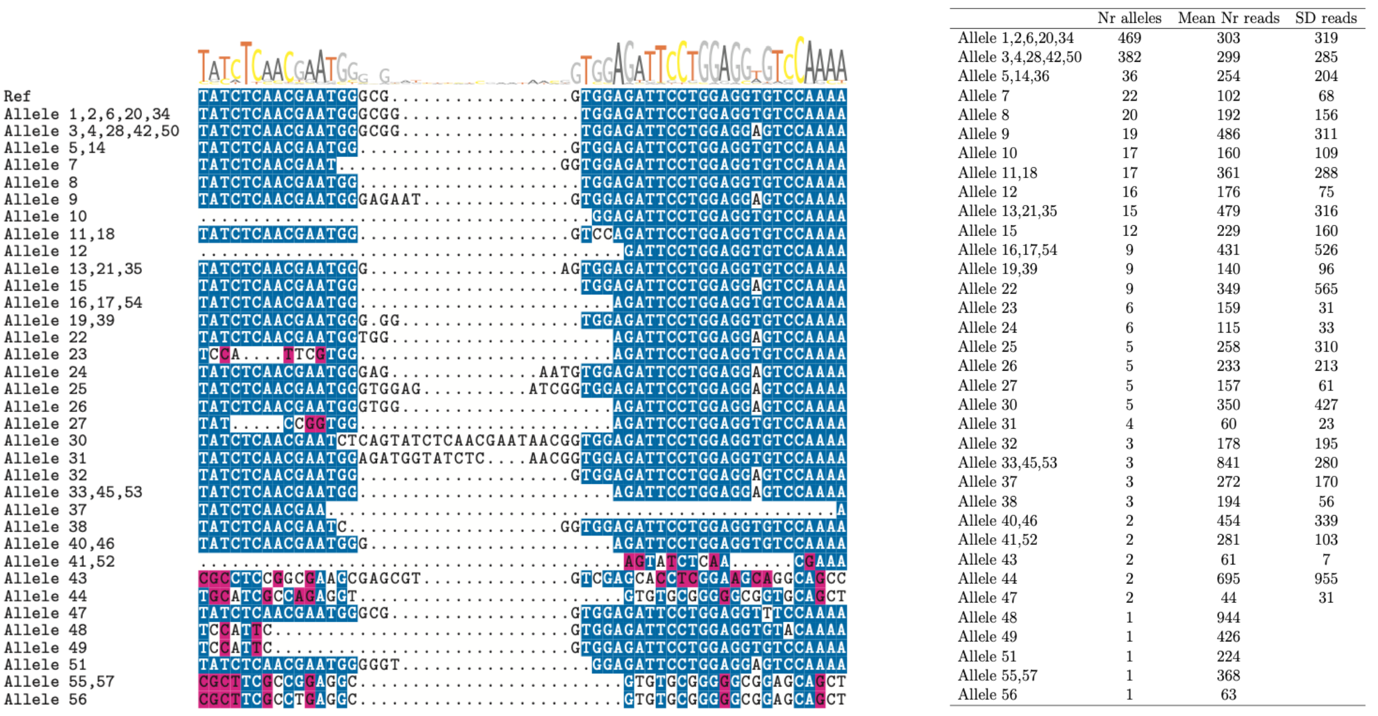
*

1. *pomca*

*
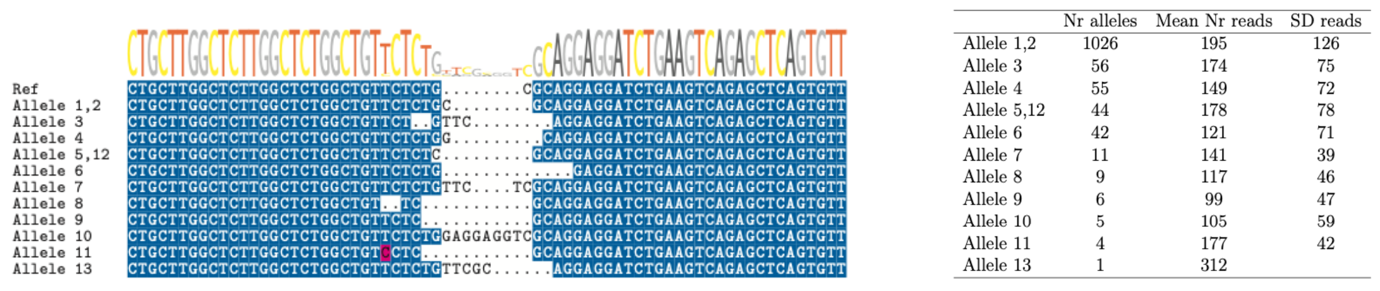
*

1. *pomcb*

*
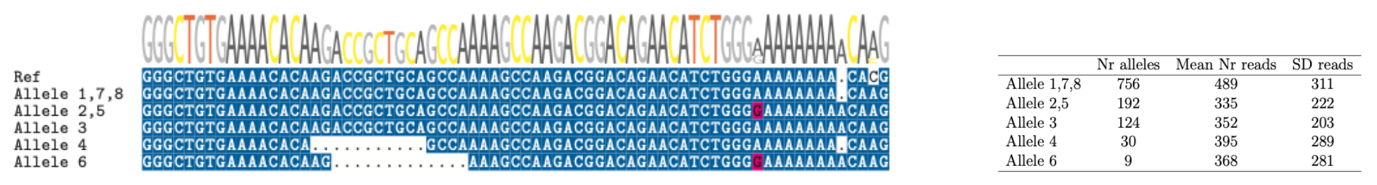
*

1. *sec16b*





1. *bdnf*

*
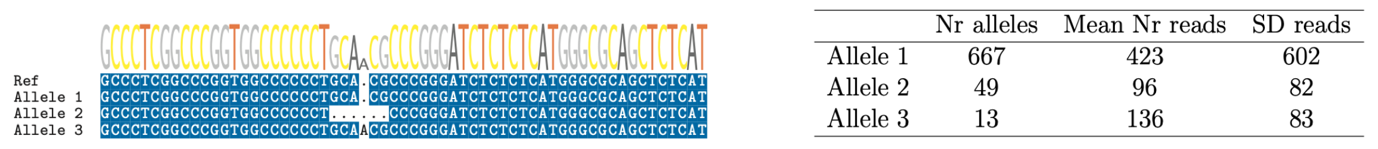
*

1. *irs1*


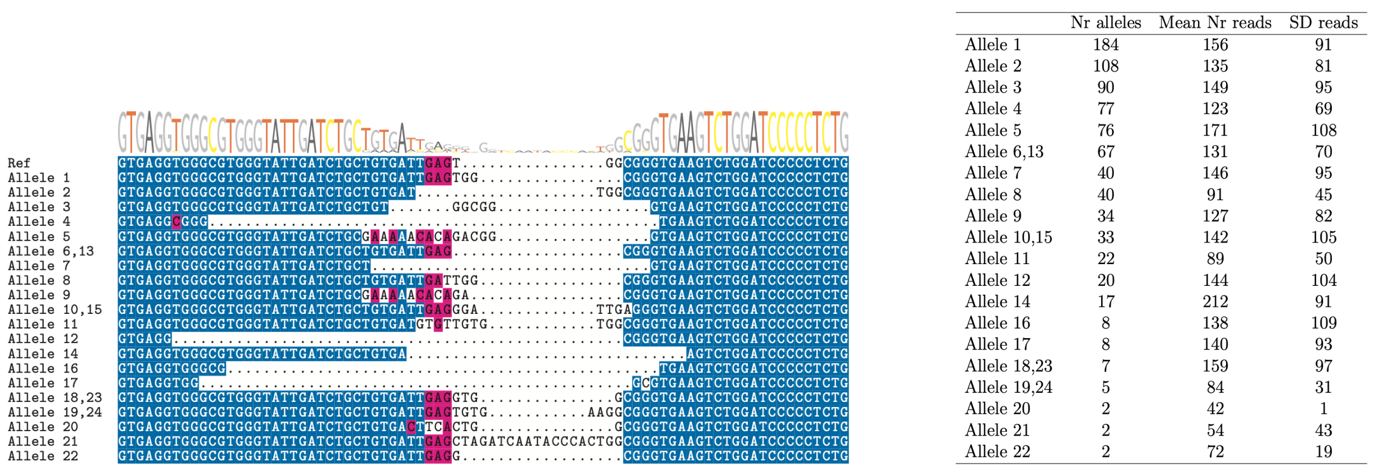


1. *irs2a*


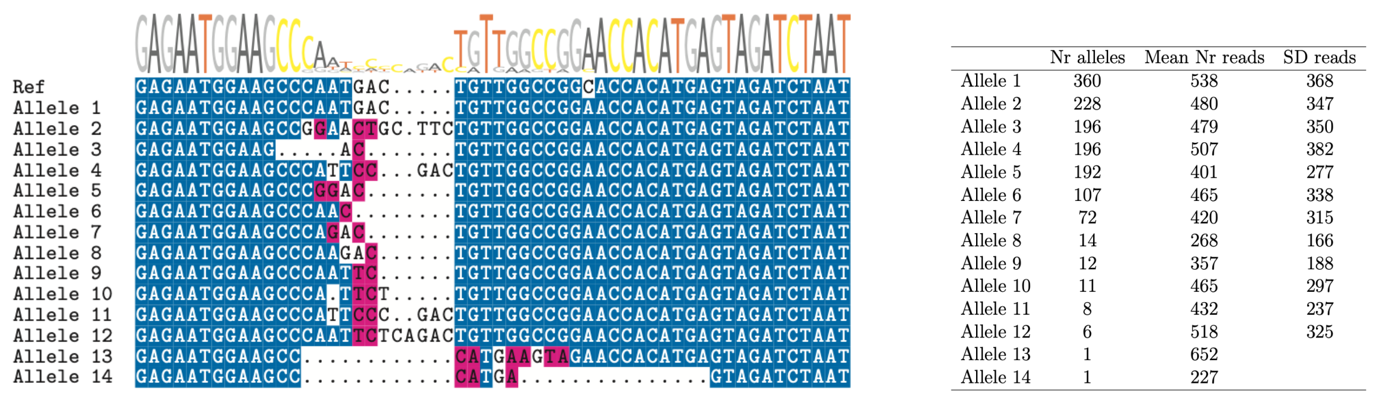


1. *irs2b*

*
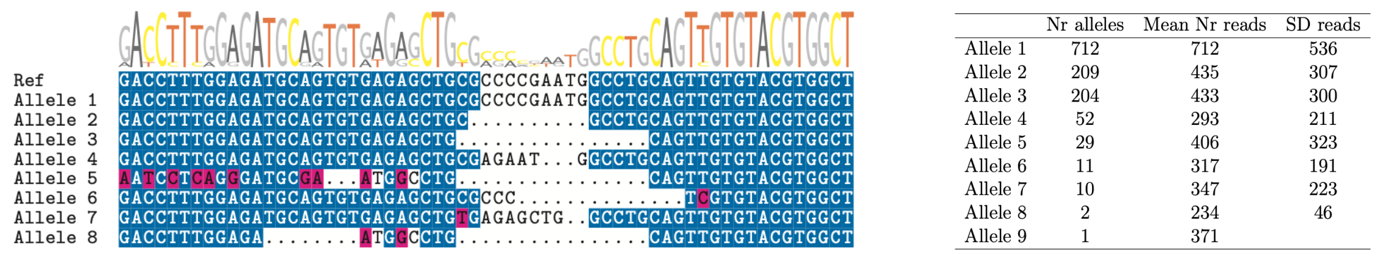
*

1. *sh2b1*


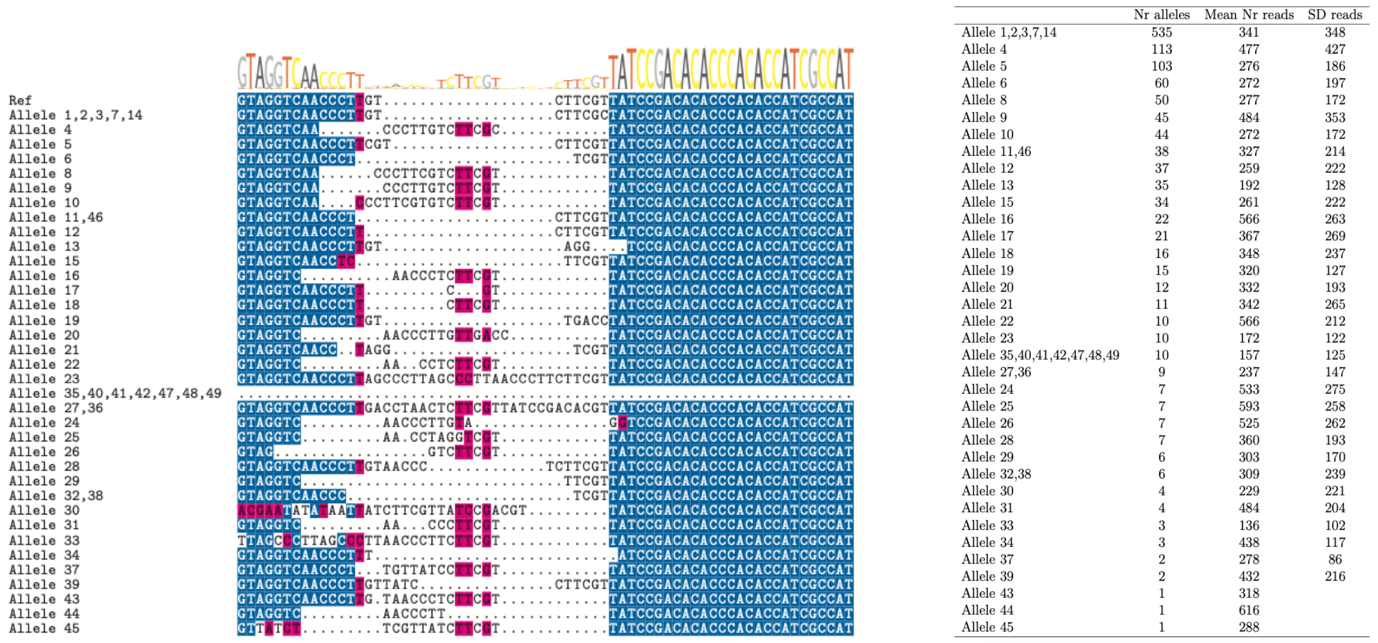


1. *sim1a*

*
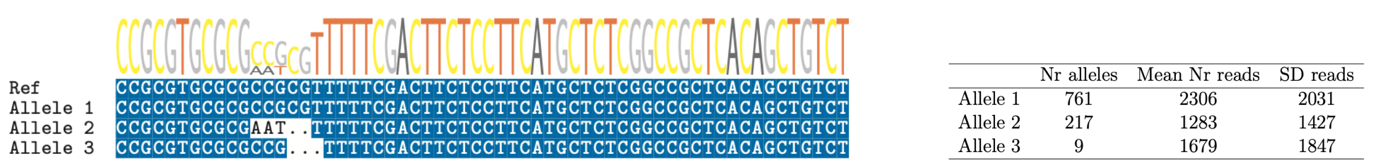
*

1. *sim1b*

*
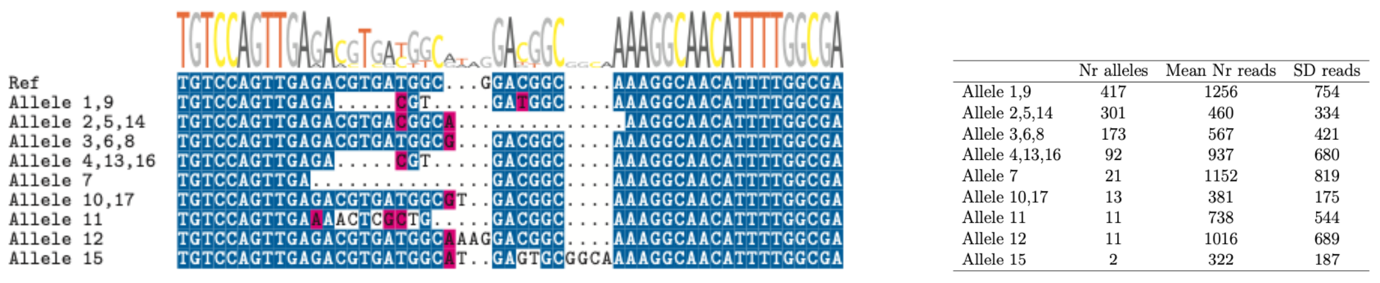
*

**Supplementary Figure 12. List of CRISPR/Cas9-induced mutations in sequenced F_1_ larvae across the 15 targeted zebrafish genes.** Alignment of reference amplicons (genome built GRCz11) and unique alleles after paired-end sequencing (2x250 bp) show CRISPR/Cas9-induced mutations. Alleles that differ by variants not attributed to CRISPR/Cas9 are grouped together and bases are color-coded as highly conserved (blue), not conserved (white), or similar (pink). Tables on the right show the number of occurrences across the sequenced zebrafish larvae (n up to 768 per multiplex), and the mean and standard deviation (SD) of the number of reads that calls are based on.

**Supplementary Figure 13. Effects of CRISPR/Cas9-induced mutations on image- and biochemistry-based cardiometabolic traits in 10-day-old F_1_ larvae, i.e., in offspring of in-crossed founders.** Open dots and error bars show effect sizes and 95% confidence intervals for each additional mutated allele weighted by the predicted impact of mutations on protein function; filled dots and error bars show effects and 95% confidence intervals for larvae with frameshift and/or premature stop codon-introducing mutations in both alleles vs. larvae free from CRISPR/Cas9-induced mutations in the gene. Effects were adjusted for the number of mutated alleles in the other targeted genes in the same multiplex (weighted by their predicted impact on protein function), as well as for batch, tank and time of day at imaging. Results shown in red have P < 0.05.


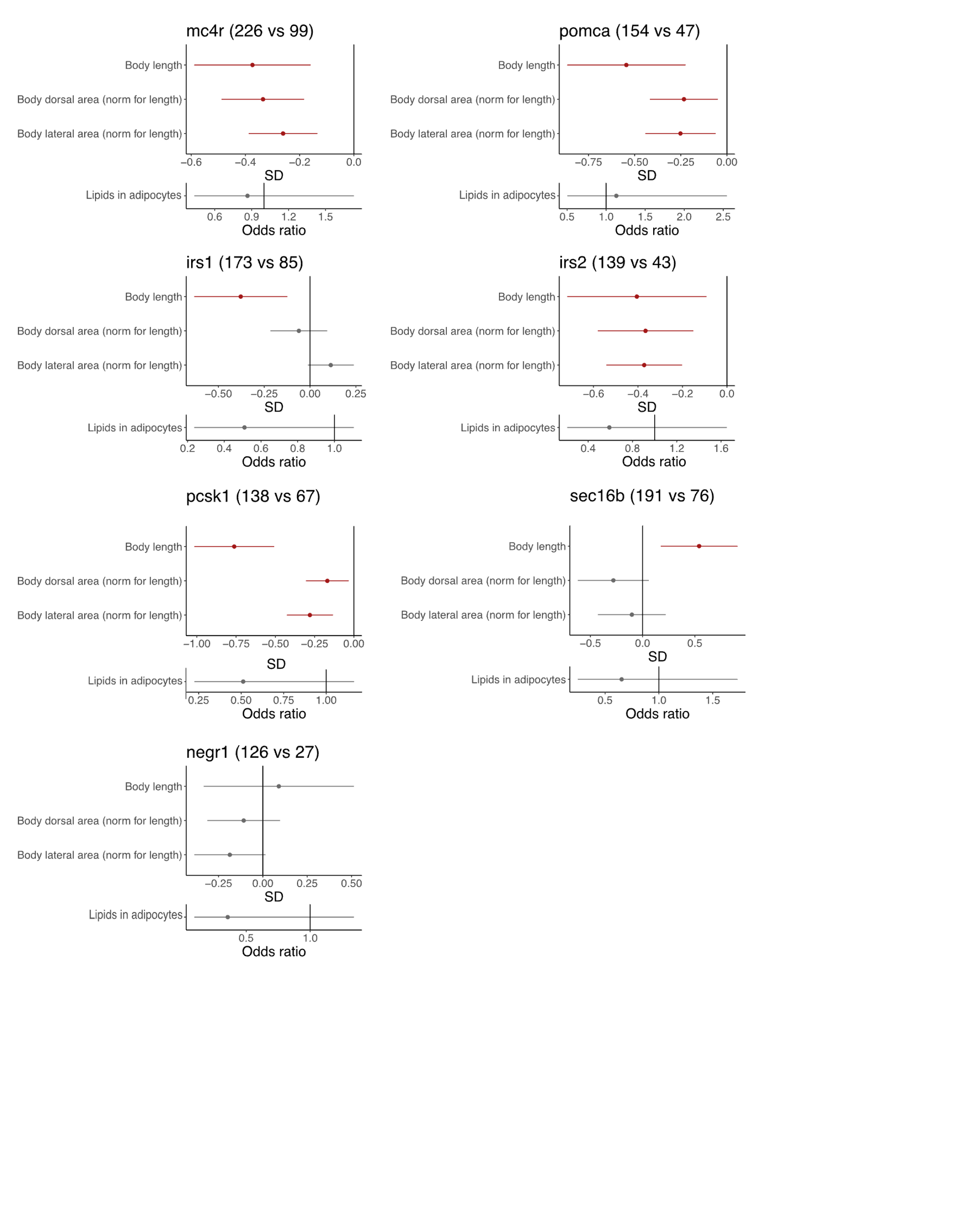
**Supplementary Figure 14. Effect of CRISPR/Cas9-induced mutations on accumulation of lipids in adipocytes in 10-day-old founders.** Dots and error bars show effect sizes and 95% confidence intervals for affected larvae vs. controls (F_0_ generation; CRISPR/Cas9 founders), as confirmed using a PCR-based fragment length analysis. Effects were adjusted for batch, tank and time of day at imaging. Numbers at the top of each plot reflect the number of affected larvae for the candidate gene (left) and the number of sibling controls (right). OR= Odds ratio.


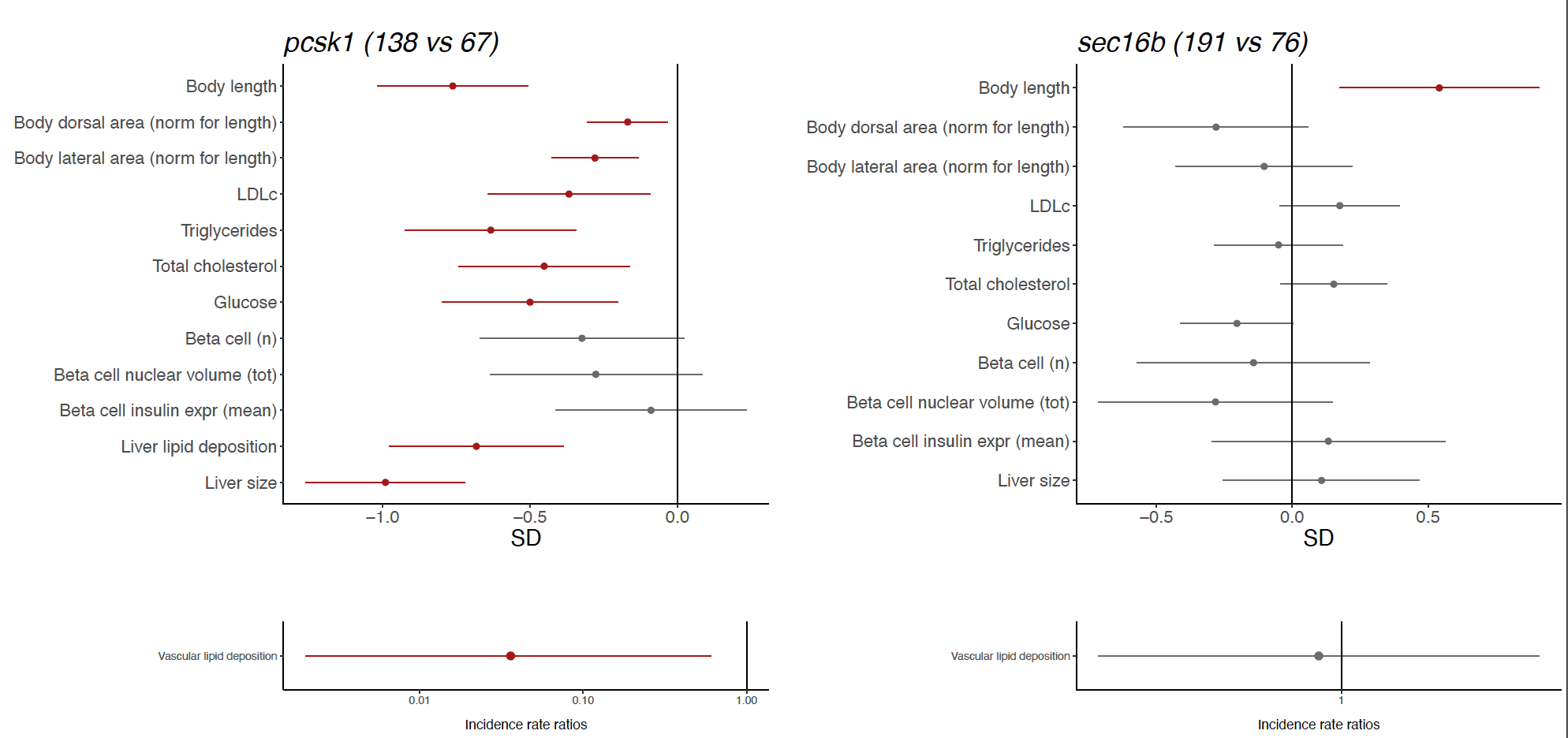


**Supplementary Figure 15. Effect of CRISPR/Cas9-induced mutations on image- and biochemistry-based cardiometabolic traits in 10-day-old founders.** Dots and error bars show effect sizes and 95% confidence intervals for affected larvae vs. controls (F_0_ generation; CRISPR/Cas9 founders), as confirmed using a PCR-based fragment length analysis. Effects were adjusted for batch, tank and time of day at imaging. Numbers at the top reflect the number of affected larvae for the candidate gene (left) and the number of sibling controls (right).


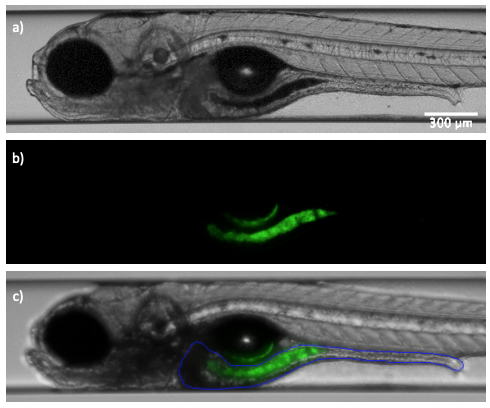


**Supplementary Figure 16. Imaged-based segmentation of food intake.** **a)** 8-day-old zebrafish larvae were overfed with standard dry food enriched with fluorescently labelled pellets for 1 h before imaging; **b-c)** the intra-abdominal fluorescence signal (green) was imaged and the signal located within the gastrointestinal tract (blue line) was automatically segmented and quantified.

**Supplementary Figure 17. Distribution of body length stratified by the presence or absence of lipid accumulation in adipocytes.** The left panel shows data for larvae with (yellow) and without (light blue) lipid deposits in at least one anatomical region (i.e., the abdominal, opercular, pectoral and cardiac regions). The right panel shows the distribution of body length, stratified by the presence (light blue) or absence (yellow) of lipid deposits in the abdominal region only. Top: multiplex 1; bottom: multiplex 2. The black vertical line (body length = 4.3mm) highlights the minimum length at which lipid deposits in adipocytes were first observed in wild-type zebrafish larvae in the literature (Imrie *et al*., 2010).


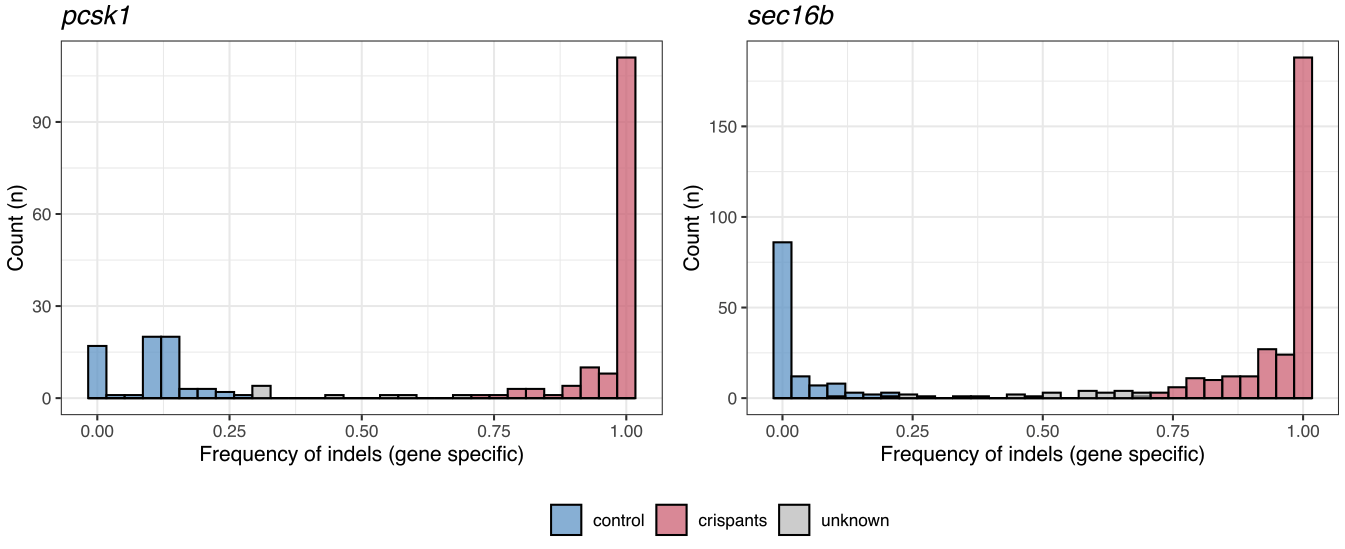


**Supplementary Figure 18. Frequency of indel mutations in larvae targeted at *kita* only (controls) or that were additionally targeted with gRNAs affecting *pcsk1* or *sec16b* (affected larvae).** Bar chart depicting the number of larvae assigned to be affected (red) or controls (blue) based on a fragment length analysis of the regions flanking the gene specific cut sites.


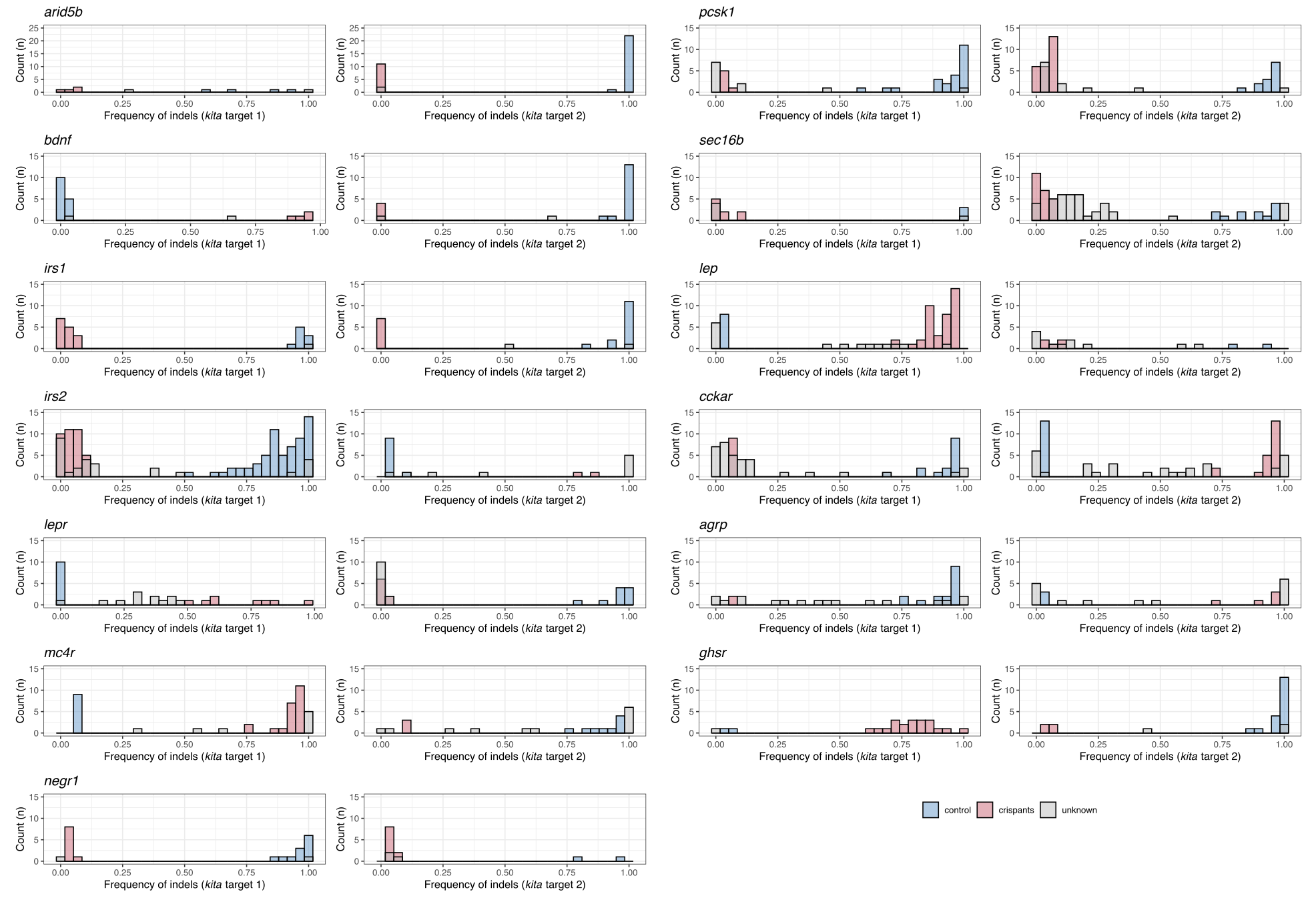


**Supplementary Figure 19. Frequency of indel mutations of larvae targeted at *kita* only (controls) or additionally targeted with gRNAs affecting orthologues of one of 13 human genes per larva (affected larvae).** The bar chart shows the number of larvae assigned to be affected (red) or controls (blue) based on a fragment length analysis of the regions flanking the *kita* target sites 1 or 2.
